# Metabolome × Microbiome Changes Associated with a Diet-Induced Reduction in Hepatic Fat among Adolescent Boys

**DOI:** 10.3390/metabo13030401

**Published:** 2023-03-08

**Authors:** Catherine C. Cohen, Helaina Huneault, Carolyn J. Accardi, Dean P. Jones, Ken Liu, Kristal M. Maner-Smith, Ming Song, Jean A. Welsh, Patricia A. Ugalde-Nicalo, Jeffrey B. Schwimmer, Miriam B. Vos

**Affiliations:** 1 Department of Pediatrics, School of Medicine, University of Colorado Anschutz Medical Campus, Aurora, CO 80045, USA; 2 Nutrition & Health Sciences Doctoral Program, Laney Graduate School, Emory University, Atlanta, GA 30322, USA; 3 Department of Medicine, School of Medicine, Emory University, Atlanta, GA 30322, USA; 4 Emory Integrated Lipidomics Core, School of Medicine, Emory University, Atlanta, GA 30322, USA; 5 Department of Medicine, University of Louisville School of Medicine, Louisville, KY 40202, USA; 6 Hepatobiology and Toxicology Center, University of Louisville School of Medicine, Louisville, KY 40202, USA; 7 Department of Pediatrics, School of Medicine, Emory University, Atlanta, GA 30322, USA; 8 Children’s Healthcare of Atlanta, Atlanta, GA 30322, USA; 9 Department of Gastroenterology, Rady Children’s Hospital San Diego, San Diego, CA 92123, USA; 10 Department of Pediatrics, School of Medicine, University of California, San Diego, CA 92093, USA

**Keywords:** sugar, fatty liver disease, obesity, pediatric, liquid chromatography-mass spectrometry

## Abstract

Dietary sugar reduction is one therapeutic strategy for improving nonalcoholic fatty liver disease (NAFLD), and the underlying mechanisms for this effect warrant further investigation. Here, we employed metabolomics and metagenomics to examine systemic biological adaptations associated with dietary sugar restriction and (subsequent) hepatic fat reductions in youth with NAFLD. Data/samples were from a randomized controlled trial in adolescent boys (11–16 years, mean ± SD: 13.0 ± 1.9 years) with biopsy-proven NAFLD who were either provided a low free-sugar diet (LFSD) (*n* = 20) or consumed their usual diet (*n* = 20) for 8 weeks. Plasma metabolomics was performed on samples from all 40 participants by coupling hydrophilic interaction liquid chromatography (HILIC) and C_18_ chromatography with mass spectrometry. In a sub-sample (*n* = 8 LFSD group and *n* = 10 usual diet group), 16S ribosomal RNA (rRNA) sequencing was performed on stool to examine changes in microbial composition/diversity. The diet treatment was associated with differential expression of 419 HILIC and 205 C_18_ metabolite features (*p* < 0.05), which were enriched in amino acid pathways, including methionine/cysteine and serine/glycine/alanine metabolism (*p* < 0.05), and lipid pathways, including omega-3 and linoleate metabolism (*p* < 0.05). Quantified metabolites that were differentially changed in the LFSD group, compared to usual diet group, and representative of these enriched metabolic pathways included increased serine (*p* = 0.001), glycine (*p* = 0.004), 2-aminobutyric acid (*p* = 0.012), and 3-hydroxybutyric acid (*p* = 0.005), and decreased linolenic acid (*p* = 0.006). Microbiome changes included an increase in richness at the phylum level and changes in a few genera within *Firmicutes*. In conclusion, the LFSD treatment, compared to usual diet, was associated with metabolome and microbiome changes that may reflect biological mechanisms linking dietary sugar restriction to a therapeutic decrease in hepatic fat. Studies are needed to validate our findings and test the utility of these “omics” changes as response biomarkers.

## 1. Introduction

Nonalcoholic fatty liver disease (NAFLD) is now the most common chronic liver disease in children, especially in certain subgroups such as children with obesity, males, and Hispanic/Latinos [1,2]. NAFLD can progress to cirrhosis and end-stage liver disease, and is associated with cardiometabolic abnormalities such as insulin resistance (IR), hypertension, and dyslipidemia [3,4,5]. Therefore, early diagnosis and effective treatments are imperative for preventing the progression of co-morbidities.

Currently, no recommended medications or supplements exist for pediatric NAFLD beyond lifestyle modification for general weight loss, which has been shown to have moderate success in reducing hepatic fat and alanine aminotransferase (ALT) [6,7,8,9,10,11,12]. Dietary sugar restriction has been proposed as a particularly effective therapeutic strategy for NAFLD. One short-term (9 day) intervention study in 41 children (9–18 years) with obesity showed that dietary restriction of the free sugar fructose (~4% of total energy intake (TEI)) was associated with improvements in hepatic fat (from 7.2% to 3.8%), hepatic de novo lipogenesis and insulin kinetics [13]. Aligning with this, we recently conducted an 8-week, randomized, controlled intervention study in 40 adolescent boys (11–16 years) with NAFLD to test the effect of dietary free sugar restriction on hepatic fat, and found that the diet treatment group achieved a significantly greater reduction in hepatic fat (from 25% at baseline to 17% at week 8) compared to usual diet (21% to 20%) [14]. Importantly, this reduction in hepatic fat was independent of weight loss, suggesting that sugar restriction may influence NAFLD outcomes through mechanisms beyond calorie restriction and energy balance; this warrants further investigation.

Advances in “omics” technologies allow us to evaluate systemic biological alterations related to health, disease, and therapeutics. High-resolution metabolomics (HRM) can be used to examine the metabolic pathway alterations associated with hepatic fat and the related cardiometabolic dysfunction. As shown in several cross-sectional studies [15,16,17,18,19,20,21], the metabolome of children with NAFLD is often characterized by elevated branched chain and aromatic amino acids, glutamate, and short-chain acylcarnitines, decreased glycine and glutamine, and disturbances in lipid metabolism, especially glycerophospholipids. The microbiome has also gained interest as a pathogenic factor in NAFLD, which may interact with the metabolome via the gut-liver axis, given its role in nutrient digestion and absorption, immune function, and gut barrier integrity. Indeed, cross-sectional studies have found that children with NAFLD, compared to healthy children, have increased intestinal permeability [21] and alterations in gut microbiota composition, including lower alpha diversity [22], as well as differential abundance of specific microbial species particularly within *Bacteroidetes*, *Firmicutes*, and/or *Actinobacteria* [22,23,24,25]. Data are lacking, however, from intervention studies in pediatric NAFLD examining changes in these metabolome and microbiome alterations due to treatment. Such prospective studies could provide needed insights into the responsiveness of underlying disturbances and/or identify novel response biomarkers.

Therefore, the objective of this study was to evaluate both metabolome and microbiome changes associated with a diet treatment-induced hepatic fat reduction in children with NAFLD. To achieve this, we performed HRM on fasting plasma collected at baseline and study completion from 40 adolescent boys with NAFLD who participated in the randomized, controlled intervention study mentioned above that tested the effect of a low free-sugar diet (LFSD) compared to a usual diet for 8 weeks [14]. In a sub-sample of 18 participants, we also performed 16S rRNA sequencing on stool at baseline and at week 8. Our hypothesis was that amino acid and lipid alterations detected by metabolomics would be normalized and the diversity and composition of the gut microbiota would be improved with the LFSD diet treatment.

## 2. Materials and Methods

### 2.1. Study Design

The parent study was an 8-week, randomized, controlled dietary treatment study conducted in 40 adolescent boys (ages 11–16 years) with NAFLD. A detailed summary of the study design, dietary treatment, and main findings of the parent study, as well as baseline characteristics of the sample, have been reported elsewhere [14].

Briefly, eligibility criteria included a clinical-pathological diagnosis of NAFLD by liver biopsy, hepatic fat > 10% based on magnetic resonance imaging-proton density fat fraction (MRI-PDFF), ALT > 45 U/L, and current sugar-sweetened beverage consumer (defined as ≥3, 8 fluid ounce drinks/week). Exclusion criteria included history of diabetes or other chronic liver disease, history of significant alcohol use, or chronic use of medications known to cause hepatic steatosis or steatohepatitis. Participants were randomized to either the treatment group, which was provided a low free-sugar version of their habitual diet (goal: <3% of TEI from free sugars) for 8 weeks, or the control group, which consumed their usual diet for 8 weeks. The primary outcome of interest was change in hepatic fat measured by MRI-PDFF from baseline to week 8.

**Figure 1:**
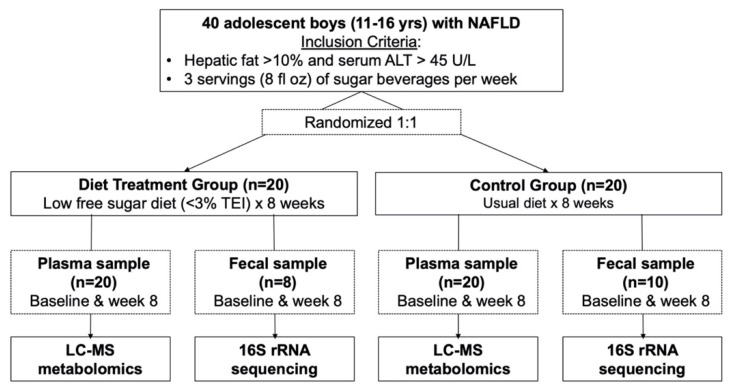
Workflow for participant inclusion in the present study. Metabolomics analysis was performed by liquid-chromatography mass spectrometry (LC-MS), and microbiome analysis was performed by 16S ribosomal RNA (rRNA) sequencing. Abbreviations: ALT, alanine aminotransferase; TEI, total energy intake; LC-MS, liquid chromatography mass spectrometry; rRNA, ribosomal RNA.

As previously reported [14], provision of the LFSD resulted in a clinically significant reduction in hepatic fat (from 25% at baseline to 17% at week 8), compared to usual diet (from 21% at baseline to 20% at week 8). The LFSD treatment group, compared to the usual diet control group, also experienced significantly greater improvements in ALT [14], as well as other metabolic markers, including hepatic de novo lipogenesis (DNL) [26]. The present study aimed to build on these prior findings and specifically assess the differential metabolome and microbiome changes that occurred in the LFSD treatment group compared to the usual diet control group, in parallel with the previously reported reduction in hepatic fat. A summary of the workflow used in the study is shown in Figure 1. Written informed consent was obtained from a parent or guardian, and assent was obtained from the adolescent participants. Ethics approval was obtained from the institutional review boards of the University of California San Diego, and Emory University.

### 2.2. High-Resolution Metabolomics

Blood samples were drawn at baseline and week 8 after an overnight fast. Plasma was collected in EDTA-coated tubes, processed immediately, and stored at −80 °C. HRM was performed on stored plasma samples using established liquid chromatography-mass spectrometry (LC-MS) methods by the Emory Clinical Biomarkers Laboratory [27,28]. Briefly, plasma samples were randomized prior to analysis and analyzed in batches of 40 using a dual chromatography platform. The platform consisted of hydrophilic interaction liquid chromatography (HILIC) with positive electrospray ionization (ESI) and reverse phase C18 chromatography with negative ESI, with detection by ultra-high resolution mass spectrometry (Q-Exactive HF Orbitrap, Thermo Scientific, San Jose, CA, USA). Each batch included pooled human plasma (QStd-3) at the beginning, middle, and end. Raw data were extracted and aligned by an xMSanalyzer [29] with apLCMS [30], and batch correction was performed using ComBat [31]. The result was a two-dimensional ‘feature table’ consisting of 9865 HILIC/+ESI and 7280 C18/ESI−chemical features, defined by accurate mass-to-charge ratio (*m*/*z*) and retention time (RT), and ion abundance in each sample, which are referred to as *m*/*z* features hereafter. To minimize clustering effects by study site, both datasets were normalized by site using ComBat [31]. Other data processing steps included filtering if missing in >80% of samples (6259 HILIC/+ESI and 4009 C18/−ESI *m*/*z* features were retained), log-transformation, and quantile normalization. Any remaining missing values were assumed to be below the detection limit and were imputed with half the feature minimum. We then calculated change values (week 8-baseline) using log-transformed intensities of each *m*/*z* feature for later analyses.

### 2.3. 16S rRNA Sequencing

Fecal samples were collected by a sub-sample of participants enrolled at Emory University. Participants collected the samples at home using sterile tubes prior to the baseline and week 8 study visits, and were stored at −80 °C until used. Microbial genomic DNA was extracted from frozen fecal samples using a DNeasy PowerSoil kit (Cat#:12888-100, Qiagen, Germantown, MD, USA) according to the manufacturer’s instructions. Libraries were prepared following Illumina’s 16S library preparation guide and Illumina’s Nextera Index Kit (FC-121-1012). Microbial genomic DNA was quantified using a Qubit 2.0 Fluorometer. The 16S variable region for each sample was amplified using 12.5 ng of microbial genomic DNA. Sequencing was done at the Genomics core at the University of Louisville using a Nano-300 cycle test chip (MS-103-1001) to confirm sample concentration, followed by Illumina MiSeq Reagents kit v3 (600 cycles) (MS-102-3003) at 9 pM and 30% PhIX. Quality control of raw sequence files was performed using FastQC (v0.10.1), and data were trimmed using trimmomatic (v0.33) due to lower quality values for bases at the end of samples. Data were then further analyzed using the QIIME 2 pipeline [32,33]. Briefly, sequences reads were demultiplexed and denoised, and assigned to operational taxonomy units (OTUs) at 97% similarity to the clustered Greengenes database [34]. To account for differences in raw sequences between samples, all taxonomic tables were normalized and log-transformed using the following formula: Log10 [((Raw count in sample_i_/# of sequences in samplei) × Average # of sequences per sample) + 1]. Similar to metabolomics data, we calculated change values (week 8-baseline) for each taxa using log-normalized abundances. This was performed on taxa present in >25% of samples to limit spurious associations. After this restriction, 56 OTUs, 38 genera, 22 families, nine classes, nine orders, and four phyla remained.

### 2.4. Other Relevant Assessments

A variety of clinical, laboratory, and dietary assessments were performed at baseline and week 8, as previously described [14]. Notably, fasting blood was collected to assess various laboratory markers, including liver enzymes [ALT, aspartate aminotransferase (AST), and gamma-glutamyl transferase (GGT)], fasting glucose and insulin, and blood lipids. Anthropometric assessments (height, weight, and waist circumference) were also performed twice at each visit and averaged. To assess dietary intakes, three, 24-h dietary recalls (2 weekday and 1 weekend day) were collected using the Nutrition Data System for Research (version 2015, University of Minnesota, Minneapolis, MN, USA). Physical activity was not assessed, but participants were asked not to make any major changes to their physical activity routines during the study.

### 2.5. Statistical Analysis

Differential changes in the plasma metabolome were assessed using linear regression models with the treatment group as the independent variable and the change value for each *m*/*z* feature as the dependent variable. We also adjusted each model for baseline values for each *m*/*z* feature. False discovery rate (FDR)-adjusted q-values were calculated based on the Benjamini-Hochberg method to account for multiple testing [35]. Differentiating *m*/*z* features were entered into pathway analysis using Mummichog v2.0.6-beta (available at: http://mummichog.org/; accessed 29 June 2020) [36]. Enriched pathways were selected based on *p* < 0.05 in permutation-based testing and an overlap size ≥ 2 (i.e., at least two differentiating *m*/*z* features were enriched in the pathway). To assess differential changes in microbial composition and diversity, we first assessed beta-diversity by performing principal coordinate analysis (PCoA) based on Bray-Curtis distance of the log-normalized abundances using the “capscale” function of the R package *vegan* [37]. We next rarefied raw counts using a subsample size equal to the sample with the fewest reads at each taxonomic level using the “rrarefy” function of *vegan*, and assessed alpha diversity at each taxonomic level using the Shannon diversity index, inverse Simpson index, and richness; evenness was calculated as the Shannon Index divided by the natural log of richness. This process was repeated 10 times, and the average value was used. Similar to the metabolomics data, we constructed baseline-adjusted linear models to assess differences between groups in change values for log-normalized abundances and diversity measures. Lastly, we examined correlations between differentiating *m*/*z* features and differentiating taxa from 16S rRNA sequencing using Kendall’s rank correlations. All analyses were carried out in RStudio (v3.5.3). Figures were created using the R package *ggplot2* [38].

### 2.6. Metabolite Annotation and Quantification

Differentially expressed *m*/*z* features were annotated using xMSannotator, a software package that matches accurate masses to common positive and negative ion mode adducts using the Human Metabolome Database (HMDB) [39], and scores all matches from 0 (accurate mass match only) to 3 (high confidence match) based on a multifactorial algorithm [40]. Annotations having an *m*/*z* and retention time of adducts, previously confirmed by comparing ion dissociation and elution time to reference standards [41], were considered Level 1 (“confirmed”) compounds according to criteria described by the Metabolomics Standard Initiative (MSI) [42]. For all others, annotations with a score of 2–3 in xMSannotator were Level 2 (“putative”) compounds, and annotations with no match or a score of 0–1 in xMSannotator were Level 4 (“unknown”) compounds according to MSI criteria. ClassyFire software [43] was used to group metabolites by compound class for organizational purposes. We next used reference standardization methods to calculate concentrations of selected metabolites with confirmed (Level 1 MSI) identities. Details of this technique are reported elsewhere [41,44]. Briefly, concentrations were calculated using single point calibration by multiplying the ion abundance for each metabolite by response factors determined by dividing the known concentration of the metabolite in Q-std3 by its ion intensity in Q-std3. For quality control purposes, the calculated concentrations of each quantified metabolite were compared to previously reported values in HMDB.

## 3. Results

The mean age of participants was 13.0 ± 1.9 years. The majority of participants were of Hispanic ethnicity (95%) and classified with overweight or obesity (98%). Eleven participants (28%; four treatment participants and seven control participants) were diagnosed with biopsy-proven NASH at baseline. As previously described [14], most baseline characteristics were similar in the LFSD treatment group compared to control group, including BMI (mean ± SD: 33.7 ± 5.6 vs. 32.3 ± 6.3 kg/m^2^, respectively) and free-sugar intake (10% TEI vs. 11% TEI, respectively). An exception was that average hepatic fat (MRI-PDFF) was higher in the diet treatment group compared to control group at baseline (mean ± SD: 25 ± 11% vs. 21 ± 8%, respectively).

After the 8-week treatment, free-sugar intake decreased to <1% TEI at week 8 in the diet treatment group, compared to 10% TEI at week 8 in the control group. In parallel, the diet treatment was associated with a greater mean decrease in hepatic fat from baseline to week 8 (25% to 17%, respectively) than the control diet (21% to 20.0%, respectively) [14].

Characteristics of the sub-sample, who also provided fecal samples for 16S rRNA sequencing, are shown in Table S1. Overall, characteristics of this sub-sample were similar compared to the full sample and there were no notable differences in any traits between treatment groups.

### 3.1. Metabolome Changes Associated with the Low-Sugar Diet Treatment

A total of 419 *m*/*z* features from HLIC/+ESI and 205 from C18/−ESI were differentially changed in the treatment compared to control group based on raw *p* < 0.05 in linear regressions adjusted for baseline. Among these, 180 *m*/*z* features (122 from HILIC/+ESI and 56 from C18/−ESI) were putatively annotated (Level 2) or confirmed (Level 1) based on MSI criteria (Table S2). We summarize the mean change values for differentially changed, confirmed (Level 1) *m*/*z* features in Table 1.

**Figure 2:**
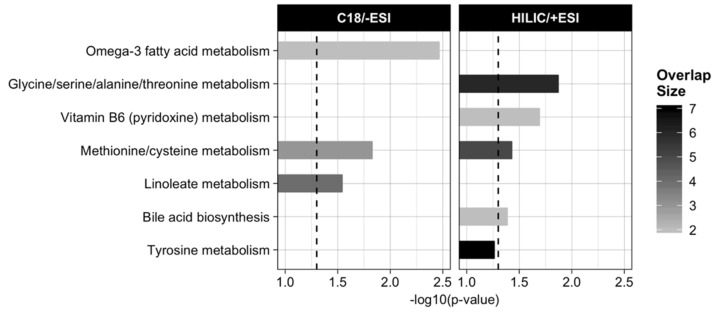
Results from pathway analysis in Mummichog of the *m*/*z* features that were differentially changed in the treatment group vs. control group, only showing pathways with *p* < 0.05 in permutation-based testing (indicated by horizontal dotted line) and with an overlap size ≥ 2 features. Abbreviations: ESI, electrospray ionization; HILIC, hydrophilic liquid interaction chromatography.

No differences remained significant after adjusting for multiple testing. Therefore, to prioritize the most biologically relevant findings, we performed untargeted pathway analysis in Mummichog based on the raw *p* < 0.05 cut-off to identify differentiating features. This revealed significant enrichment in pathways involved in amino acid metabolism, including methionine/cysteine, tryptophan, and glutamate metabolism, fatty acid metabolism, including omega-3 fatty acid metabolism and linoleate metabolism, bile acid metabolism, and vitamin B6 (pyridoxine) metabolism (all *p* < 0.05) (Figure 2).

**Figure 3:**
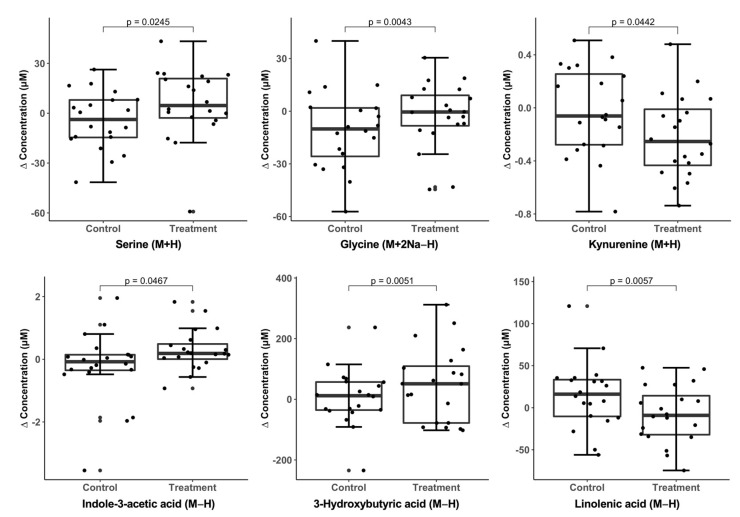
Boxplots showing change values for select quantified metabolites by diet treatment group. *p*-values were calculated using linear regression adjusted for baseline values. Metabolite concentrations were calculated using the reference standardization technique [41]. Reference levels for each metabolite in the Human Metabolome Database are shown in Table S3.

To further interpret the findings in a clinical context, reference standardization was used to estimate absolute concentrations for a subset of identified, differentiating metabolites. The mean concentrations by treatment group and time are shown in Table S3. Representative of the enriched amino acid pathways above, the treatment compared to control group had increased levels of serine, glycine, acetylycine, and 2-aminobutyric acid after 8 weeks, as well as decreased kynurenine and increased indole-3-acetic acid (two tryptophan-related metabolites) (Figure 3, *p* < 0.05). Representative of the enriched fatty acid pathways, there was an increase in the ketone body 3-hydroxybutric acid and a decrease in linolenic acid in the treatment compared to control group (Figure 3, *p* < 0.05).

### 3.2. Microbiome Changes Associated with the Low-Sugar Diet Treatment

**Figure 4:**
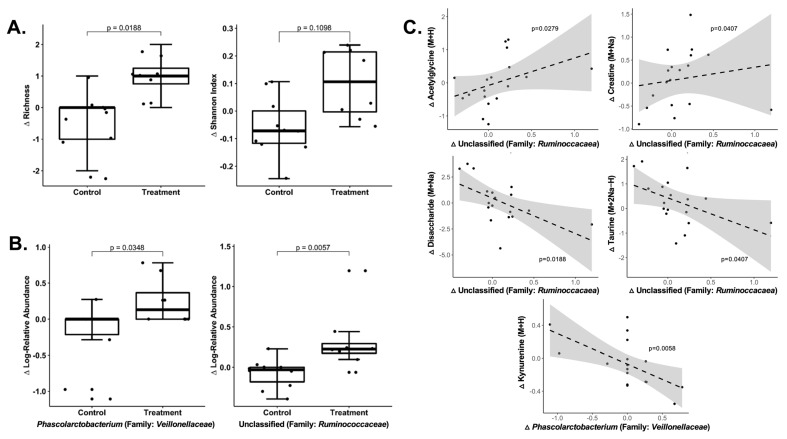
Changes in rarefied diversity measures and microbial composition by treatment group and correlations between differentially expressed metabolites and genera. (**A**) Boxplots of change values for richness and Shannon diversity index at the phylum level. (**B**) Boxplots of change values for genera that were differentially changed in the treatment group versus control group (*p* < 0.05 in baseline-adjusted linear regression). No results were significant after adjusting for multiple testing. (**C**) Scatterplots showing significant correlations between differentially changed metabolites and genera (*p* < 0.05, FDR-adjusted q < 0.20 based on Kendall’s correlation). Only showing results for metabolites (y-axis) that were confirmed (Level 1) based on MSI criteria.

We used 16S rRNA sequencing to assess gut microbiota at baseline and week 8 in a sub-sample of 19 participants (all residing in Atlanta, GA, USA). A total of 312,917 reads were obtained from 37 fecal samples (mean 8457 reads/sample). One participant in the treatment group was missing a fecal sample at week 8 and was excluded from further analyses. PCoA analysis was performed using Bray-Curtis dissimilarity. Based on the first multidimensional scaling (MDS) axis, there were significant correlations between time points (baseline and week 8) at the family and genus level as expected (Figure S1). However, MDS ordination showed that there were no significant differences between groups for change values for the first or second MDS axes (Figure S2, Table S4). After rarefying the data, we found that richness increased in the treatment versus control group at the phylum level (*p* = 0.02, Figure 4) and trended toward being increased at the class level (*p* = 0.09) (Table S5). We next assessed changes in microbial abundance and found that the log-normalized abundance of two genera, which were one unclassified genus from family *Ruminococcaceae* (*p* = 0.006) and *Phascolarctobacterium* from family *Veillonellaceae* (*p* = 0.035) (Figure 4), and three OTUs, including *Ruminococcus bromii* (*p* = 0.026), were increased in the treatment compared to control group after 8 weeks (Table S6). However, similar to the metabolomics results, no findings were significant after FDR correction.

### 3.3. Integrative Analysis of Metabolome and Microbiome Changes

In an exploratory analysis, we examined correlations between the differentially changed *m*/*z* features (419 HILIC/+ESI and 205 C18/−ESI, based on raw *p* < 0.05) and differentially changed genera (based on raw *p* < 0.05) using Kendall’s rank correlations. This revealed significant correlations between 108 *m*/*z* features (77 HILIC/+ESI and 31 C18/−ESI) and the unclassified genera in *Ruminococcaceae*, and between 118 *m*/*z* features (98 HILIC/+ESI and 20 C18/−ESI) and *Phascolarctobacterium* (raw *p* < 0.05). In Figure 4, we plotted scatterplots for select metabolome x microbiome correlations (selected based on confirmed Level 1 metabolite identity and a correlation with *p* < 0.05 and FDR-adjusted q < 0.20). Acetylglycine (r = 0.38, *p* = 0.03, q = 0.18) and creatine (r = 0.35, p = 0.04, q = 0.19) were positively correlated, and disaccharide (r = −0.41, *p* = 0.02, q = 0.19) and taurine (r = −0.46, *p* = 0.008, q = 0.14) were negatively correlated with the unclassified genera in *Ruminococcaceae* (Figure 4). In addition, kynurenine (r = −0.52, *p* = 0.006, q = 0.14) was negatively correlated with *Phascolarcotobacterium* (Figure 4).

### 3.4. Sample Size Estimations for Future Studies

Post-hoc analyses were performed to guide the design of future studies testing metabolome × microbiome responses to diet treatment in pediatric NAFLD. Specifically, using the data from this pilot study, we estimated the sample sizes that would be needed to detect significant changes in our metabolome and microbiome data after FDR correction using simulated datasets (Analysis code available at: https://github.com/FarnazFouladi/PowerAnalysis/blob/master/PowerEstimation.R; accessed 22 January 2021). Based on 6259 HILIC/+ESI *m*/*z* features after data pre-processing, 10% true positives, and the smallest observed effect size with raw *p* < 0.05 (d = 0.6596, estimated using the *esc* package in R), we estimated that a sample size of 57 participants per group would be needed to achieve an average power > 80% after FDR correction. For the microbiome data, we performed the analysis at the genus level, based on 57 none-rare genera (prevalence > 10%) and the smallest borderline effect size with raw *p* < 0.10 (d = 0.9098), and estimated that 31 participants per group would be needed to achieve an average power > 80% after FDR correction.

## 4. Discussion

We examined the systemic biological changes, assessed by untargeted metabolomics and 16S rRNA metagenomics, linked to a therapeutic reduction in hepatic fat measured by MRI in youth with NAFLD. The findings build upon prior cross-sectional studies examining ‘omics’ disturbances in pediatric NAFLD and provide insight into which disturbances may be most responsive to reductions in dietary sugar and/or hepatic fat. In the plasma metabolome, we found that the predominant shifts were related to amino acid and lipid metabolic pathways and may reflect the normalization of critical biological processes. In the gut microbiome, we found an increase in richness at higher phylogenetic levels and differential changes in Firmicutes taxa. We also identified potentially novel correlations between differentially changed metabolites and taxa, supporting a link between the circulating metabolome and the composition of the gut microbiota. Although most associations were no longer statistically significant after correcting multiple testing, and should be interpreted with caution, these pilot results still provide critical information for the design of future intervention studies in pediatric NAFLD.

### 4.1. Changes in Amino Acid- and Lipid-Related Metabolites

Using a framework that coupled metabolite feature selection with untargeted pathway analysis, we identified key metabolic pathways that were enriched with differentially changed metabolites, which allowed us to prioritize the metabolomics findings with the greatest potential for biological relevance. Among these findings, both methionine/cysteine metabolism and serine/alanine/glycine metabolism were enriched with differentially changed metabolites. These pathways encompass several sub-pathways related to one-carbon metabolism, such as the methionine cycle and the trans-sulfuration pathway. Within these pathways, notable amino acid changes in the treatment group were increases in serine, glycine, and acetylglycine. A prior adult study showed that these metabolites were lower in adults with high hepatic fat [45], and their increased levels in this study may reflect improved oxidative stress and glutathione metabolism secondary to the hepatic fat reduction [46]. This is further supported by parallel increases in 2-aminobutyric acid, which has been suggested to reflect glutathione homeostasis [47], and was also inversely associated with dietary intakes of sugary foods/beverages in the Atherosclerosis Risk in Communities Study [48].

Contrary to our hypothesis, none of the aromatic or branched chain amino acids were changed due to the treatment, despite a consistent finding that these are disturbed in children with NAFLD [15,16,17,18]. There were, however, changes in tryptophan-related metabolites, including decreased kynurenine, an intermediate in tryptophan degradation, and increased indole-3-acetic acid, a microbial tryptophan metabolism. Both may be causally linked to NAFLD as aryl hydrocarbon receptor ligands, which modulate key processes involved in liver steatosis and inflammation [49,50]. Kynurenine levels in particular are determined by the activity of dioxygenase enzymes, which can be modulated by immune and/or stress-related mediators [51]. It is possible that the diet treatment normalized these processes either directly or indirectly via the microbiome, which has been shown to interact with the host immune system [52]. The latter possibility is supported by our finding that changes in kynurenine correlated with the taxa Phascolarctobacterium.

The diet treatment also induced changes in key lipid classes previously associated with NAFLD [53,54], including linolenic acid and several glycerophospholipids (Table S2). Likewise, the ketone body 3-hydroxybutyric acid was increased with the diet treatment, consistent with an adult study showing increased levels of 3-hydroxybutryic acid after a very low carbohydrate diet [55], and may reflect improved ketogenic potential and metabolic flexibility [56].

### 4.2. Changes in Microbial Diversity and Abundance

The diet treatment was also associated with a few microbiome changes, which included an increase in richness at the phylum level and changes in two genera and three OTUs within *Firmicutes*. One example was an increase in *Ruminococcus bromii*, which has been shown to be responsive to diets high in resistant starch [57], and could reflect unintended changes in other aspects of the diet beyond sugar restriction (i.e., an increase in dietary fiber) in the treatment group. At the same time, it is partially expected that our microbiome findings would be weaker than the metabolome findings given evidence that humans tend to exhibit only subtle microbiome changes in response to diet [58]. It is possible that the sub-sample size in this pilot study with microbiome data was too small to detect these subtle changes.

When we integrated the change values for the above genera with change values from the metabolomics data, we found several significant correlations; for example, the correlation between the tryptophan metabolite kynurenine and *Phascolarctobacterium* discussed above, and correlations between several amino acid metabolites and disaccharide with the unclassified genera in *Ruminococcaea*. This exploratory analysis provided insight into the information we could gain from integrative “omics” research and supports that there may be an influence of the microbiome on the metabolome changes observed, but future studies with larger sample sizes will be needed to validate these findings.

### 4.3. Limitations and Strengths

This study provides a strong foundation for characterizing the biological changes that occur with a clinically effective NAFLD intervention, but a limitation was the homogenous sample, which limits the generalizability of findings. As a pilot study, our sample size was likely under powered and may explain why no differences were significant after adjusting for multiple testing. This can be common in human metabolomics studies [59], due to the collinear nature of metabolites, which can make traditional multiple testing corrections inappropriate. We instead evaluated raw *p*-values to avoid the loss of relevant findings and type II error [60], and interpreted our findings in the context of the pathway enrichment results, but false positives are possible and larger studies are needed to confirm these findings. By using 16S rRNA sequencing, we were not able to measure microbial activity and therefore did not fully capture the effect of diet treatment on the microbiome. In addition, because the treatment group experienced changes in diet and hepatic fat over 8 weeks, we are unable to differentiate whether our findings were due to a reduction in dietary free-sugars, hepatic fat, or both. Strengths were the use of an optimized, high-resolution metabolomics approach to comprehensively measure the plasma metabolome, in parallel with the incorporation of 16S rRNA sequencing to measure the gut microbiome composition and diversity. We could, therefore, evaluate a broad spectrum of metabolic perturbations from a systems biology approach and assess both expected and novel molecular changes. Another strength was the sophisticated clinical measures used to evaluate the therapeutic effects of the intervention, including magnetic resonance imaging to assess changes in hepatic fat. In addition, the repeated measures design of the dietary treatment study allowed us to control for individual variation and assess within-person changes in metabolome and microbiome profiles over time, increasing our confidence that the changes observed were due to the intervention.

## 5. Conclusions

In summary, the findings from this integrative metabolome-by-microbiome analysis provide insight into the network of underlying biological changes that were associated with provision of a low free sugar diet for 8 weeks, and subsequent hepatic fat reduction, among adolescent boys with NAFLD. The metabolome and microbiome changes identified in the diet treatment group may represent response biomarkers related to the change in dietary free sugar content and/or hepatic fat fraction. Given the small sample size as a pilot study, larger scale studies are needed to confirm both the metabolome and microbiome findings from this study, and to disentangle the causal mechanisms at play.

## Figures and Tables

**Table 1:** Least squares (LS) mean change values and 95% CIs for select confirmed *m*/*z* features by diet treatment group.

					Control Group (n = 20)	Treatment Group (n = 20)	
Column	*m*/*z* ^b^	Time (s)	Name	Adduct	Mean Change (95% CI) ^a^	Mean Change (95% CI) ^a^	*p*-Value
HILIC/ +ESI	104.0706	82.3	2-Aminobutyric acid	M + H	0.00 (−0.15, 0.15)	0.28 (0.13, 0.44)	0.0122
	106.0499	98.3	Serine	M + H	−0.03 (−0.11, 0.05)	0.11 (0.02, 0.19)	0.0245
	118.0498	25.4	Acetylglycine	M + H	−0.15 (−0.38, 0.09)	0.21 (−0.03, 0.44)	0.0393
	120.0032	86.3	Glycine	M + 2Na-H	−0.15 (−0.24, −0.07)	0.03 (−0.06, 0.11)	0.0043
	126.022	87.6	Taurine	M + H	0.28 (0.10, 0.46)	−0.08 (−0.26, 0.10)	0.0059
	147.0764	97.8	Glutamine	M + H	0.05 (−0.02, 0.12)	−0.06 (−0.13, 0.01)	0.0304
	154.0587	84.6	Creatine	M + Na	−0.07 (−0.27, 0.13)	0.22 (0.03, 0.42)	0.0405
	166.0856	72.7	Phenylalanine	M + H	0.16 (0.07, 0.24)	0.01 (−0.08, 0.10)	0.0206
	209.092	66.5	Kynurenine	M + H	0.01 (−0.09, 0.12)	−0.14 (−0.24, −0.03)	0.0442
	269.2261	43.7	Vitamin A (Retinol)	M + H-H_2_O	0.06 (−0.04, 0.15)	−0.1 (−0.19, −0.01)	0.0196
	365.105	103.7	Disaccharide	M + Na	0.57 (−0.13, 1.28)	−0.53 (−1.23, 0.18)	0.0323
	524.3714	57.9	LysoPC(18:0)	M + H	−0.03 (−0.15, 0.10)	−0.21 (−0.34, −0.09)	0.0403
C18/ −ESI	103.04	38.9	3-Hydroxybutyric acid	M-H	−0.05 (−0.23, 0.14)	0.35 (0.16, 0.54)	0.0051
	147.0663	18.4	Mevalonic acid	M-H	−0.14 (−0.49, 0.21)	0.38 (0.03, 0.73)	0.0436
	174.0561	46.5	Indole-3-acetic acid	M-H	−0.07 (−0.31, 0.16)	0.27 (0.03, 0.50)	0.0467
	277.2173	226.8	Linolenic acid	M-H	0.26 (0.09, 0.43)	−0.09 (−0.26, 0.08)	0.0057

^a^ Mean change values and 95% CIs were calculated as least squares means from linear regression models adjusted for baseline values. ^b^ Only showing *m*/*z* features with differentially expressed change values between groups based on *p* < 0.05 and with confirmed identities based on Level 1 MSI criteria. A full list of differentially expressed *m*/*z* features with confirmed or putatively annotated identities is in Table S2. Abbreviations: HILIC, hydrophilic liquid interaction chromatography; ESI, electrospray ionization; LysoPC, lysophosphatidylcholine; MSI, Metabolomics Standard Initiative.

## Data Availability

Data available upon reasonable request. Data is not publicly available due to privacy.

## Supplementary Materials

The following supporting information can be downloaded at: https://www.mdpi.com/article/10.3390/metabo13030401/s1, Figure S1: Correlations between baseline (week 0, x-axis) and week 8 (y-axis) for the first multidimensional scaling axis (MDS) in PCoA analysis at each taxa level; Figure S2: Multidimensional scaling (MDS) ordination at each taxonomic level. Ordination based on Bray-Curtis dissimilarity; Table S1: Baseline characteristics of the sub-sample of participants (n = 19) who provided stool samples for 16S rRNA metagenomics sequencing; Table S2: Mean change values and 95% confidence intervals by treatment group for all confirmed (Level 1 MSI) or putatively annotated (Level 2 MSI) *m*/*z* features from both the HILIC/+ESI column and the C18/-ESI column; Table S3: Concentrations for quantified and confirmed metabolites that were differentially changed from baseline to week 8 in the diet treatment group compared to control group; Table S4: Least squares (LS) mean change values and 95% confidence intervals (CIs) by treatment group for the first and second multidimensional scaling (MDS) axis from PCoA analysis based on Bray-Curtis dissimilarity; Table S5: Least squares (LS) mean change values and 95% confidence intervals (CIs) by treatment group for rarefied microbial diversity measures; Table S6: Mean change values and 95% confidence intervals (CIs) by treatment group for the log-normalized relative abundance of bacteria according to each taxonomic level.
