# Supplementary material for: Metabolome × Microbiome Changes Associated with a Diet-Induced Reduction in Hepatic Fat among Adolescent Boys
Source: Metabolites. 2023 Mar 8;13(3):401. doi: 10.3390/metabo13030401 (PMC10053986; doi:10.3390/metabo13030401)
Supplement: Supplementary file 1 [file metabolites-13-00401-s001.zip › metabolites-2237426-supplementary.pdf]

Supplementary Materials:

"Metabolome x microbiome changes associated with a diet-induced reduction in hepatic fat among adolescent boys"

By:

Catherine C. Cohen[1*], Helaina Huneault[2], Carolyn J. Accardi[3], Dean P. Jones[3], Ken Liu[3], Kristal M. Maner-Smith[4], Ming Song[5,6], Jean A. Welsh[2,7,8], Patricia A Ugalde-Nicola[9,10], Jeffrey B. Schwimmer[9,10] and Miriam B. Vos[2,7,8]

Author Affiliations:

[1] Department of Pediatrics, School of Medicine, University of Colorado Anschutz Medical Campus, Aurora, CO USA

[2] Nutrition & Health Sciences Doctoral Program, Laney Graduate School, Emory University, Atlanta, GA USA

[3] Department of Medicine, School of Medicine, Emory University, Atlanta, GA USA

[4] Emory Integrated Lipidomics Core, School of Medicine, Emory University, Atlanta, GA USA

[5] Department of Medicine, University of Louisville School of Medicine, Louisville, KY USA

[6] Hepatobiology and Toxicology Center, University of Louisville School of Medicine, Louisville, KY USA

[7] Department of Pediatrics, School of Medicine, Emory University, Atlanta, GA USA

[8] Children's Healthcare of Atlanta, Atlanta, GA USA

[9] Department of Gastroenterology, Rady Children's Hospital San Diego, San Diego, CA USA

[10] Department of Pediatrics, School of Medicine, University of California, San Diego, CA USA

***Correspondence to:** Catherine.Cohen@cuanschutz.edu

Table S1: Baseline characteristics of the sub-sample of participants (n=19) who provided stool samples for 16S rRNA metagenomics sequencing

	Usual Diet Group (n=10)		Diet Treatment Group (n=9)		
**Variable**	**Mean/Median**	**SD/IQR**	**Mean/Median**	**SD/IQR**	**p-value**
Age (years)	13.3	1.83	12.44	1.59	0.294
Hepatic Fat (%)[a]	19	13.1, 25.4	27.3	22.0, 27.7	0.165
Waist circumference (cm)	109.7	15.4	95.18	37.8	0.279
Hip circumference (cm)	110.23	13.07	93.96	37.55	0.214
BMI Z-score[a]	2.43	2.10, 2.62	2.37	2.28, 2.47	0.935
ALT (U/L)[a]	92	66.0, 119.3	104	72.0, 150.0	0.513
AST (U/L)[a]	40	28.0, 53.3	51	32.0, 61.0	0.540
GGT (U/L)[a]	51.5	29.8, 75.5	58	23.0, 63.0	0.806
Glucose (mg/dL)	95.9	12.15	97.33	8.03	0.768
Insulin (mg/dL)[a]	34.4	31.0, 55.9	31.4	23.5, 57.8	0.513
HOMA-IRa	7.8	7.1, 11.5	7.9	5.7, 13.6	0.624
Total Cholesterol (mg/dL)	149.6	31.01	168.67	43.57	0.283
LDL Cholesterol (mg/dL)	98	24.15	113.44	36.48	0.287
HDL Cholesterol (mg/dL)	38	5.33	39.11	8.07	0.725
Triglycerides (mg/dL)[a]	155.5	120.3, 178.8	107	83.0, 234.0	0.653

[a] Estimates presented as Medians and IQRs due to skewed distribution

Table S2: Mean change values and 95% confidence intervals by treatment group for all confirmed (Level 1 MSI) or putatively annotated (Level 2 MSI) m/z features from both the HILIC/+ESI column and the C18/-ESI column

A) HILIC / +ESI Column								
**m/z**	**Time (s)**	**Compound Name**	**Adduct**	**MSI Level**	**Control Group Mean Δ (95% CI)**	**Treatment Group Mean Δ (95% CI)**	**p-value[a]**	**q-value[b]**
88.0393	103.1	2-Aminoacrylic acid	M+H	2	-0.12 (-0.25, 0.02)	0.14 (0.01, 0.28)	0.0090	0.6323
99.044	287.8	2-Furanmethanol	M+H	2	0.28 (-0.13, 0.7)	-0.38 (-0.79, 0.04)	0.0317	0.7318
104.0706	82.3	2-Aminobutyric acid	M+H	1	0 (-0.15, 0.15)	0.28 (0.13, 0.44)	0.0122	0.6836
105.0547	25.9	3-Hydroxybutyric acid	M+H	2	0.09 (-0.01, 0.18)	0.22 (0.12, 0.31)	0.0490	0.7419
106.0499	98.3	Serine	M+H	1	-0.03 (-0.11, 0.05)	0.11 (0.02, 0.19)	0.0245	0.7318
106.0863	228.4	Diethanolamine	M+H	2	-0.35 (-0.71, 0.01)	0.18 (-0.18, 0.53)	0.0481	0.7391
107.0855	272.3	m-Xylene	M+H	2	-0.51 (-1, -0.02)	0.27 (-0.23, 0.76)	0.0303	0.7318
108.0808	117.6	3-Ethylpyridine	M+H	2	-0.25 (-0.48, -0.01)	0.19 (-0.05, 0.43)	0.0137	0.6948
113.0961	30	4-Heptenal	M+H	2	-0.13 (-0.39, 0.13)	0.25 (-0.01, 0.5)	0.0411	0.7347
115.039	291.6	Norfuraneol	M+H	2	0.49 (0.19, 0.78)	-0.18 (-0.48, 0.11)	0.0035	0.6072
118.0498	25.4	Acetylglycine	M+H	1	-0.15 (-0.38, 0.09)	0.21 (-0.03, 0.44)	0.0393	0.7347
119.0855	242.6	Indane	M+H	2	-0.28 (-0.55, -0.01)	0.16 (-0.1, 0.43)	0.0236	0.7318
120.0032	86.3	Glycine	M+2Na-H	1	-0.15 (-0.24, -0.07)	0.03 (-0.06, 0.11)	0.0043	0.6072
121.0648	22.3	4-Hydroxystyrene	M+H	2	-0.01 (-0.13, 0.12)	0.26 (0.13, 0.39)	0.0068	0.6284
123.0652	213.7	Erythritol	M+H	2	-0.19 (-0.49, 0.11)	0.24 (-0.06, 0.54)	0.0464	0.7390
123.1168	246.4	Santene	M+H	2	-0.45 (-0.66, -0.25)	0.19 (-0.01, 0.4)	0.0001	0.3286
126.022	87.6	Taurine	M+H	1	0.28 (0.1, 0.46)	-0.08 (-0.26, 0.1)	0.0059	0.6072
127.0727	201	Melamine	M+H	2	0.1 (-0.21, 0.4)	-0.35 (-0.65, -0.04)	0.0459	0.7390
129.1274	26.8	Octanal	M+H	2	-0.08 (-0.2, 0.04)	0.13 (0.01, 0.25)	0.0167	0.7283
135.0441	19.7	Phthalide	M+H	2	-0.07 (-0.4, 0.26)	0.51 (0.18, 0.84)	0.0172	0.7283
135.1168	255	p-Cymene	M+H	2	-0.64 (-1.05, -0.24)	0.03 (-0.38, 0.44)	0.0236	0.7318
139.0754	210.4	Tyrosol	M+H	2	0.03 (-0.23, 0.28)	0.41 (0.16, 0.66)	0.0362	0.7347
140.107	20	9-Azabicyclo331nonan-3-one	M+H	2	0.55 (0.04, 1.06)	-0.21 (-0.72, 0.3)	0.0398	0.7347
147.0764	97.8	Glutamine	M+H	1	0.05 (-0.02, 0.12)	-0.06 (-0.13, 0.01)	0.0304	0.7318
148.0039	82.4	Taurine	M+Na	1	0.31 (0.11, 0.52)	-0.13 (-0.33, 0.07)	0.0041	0.6072
149.0598	212.2	Cinnamic acid	M+H	2	-0.09 (-0.38, 0.21)	0.39 (0.1, 0.69)	0.0275	0.7318
150.0914	16	Venoterpine	M+H	2	0.17 (-0.01, 0.34)	0.53 (0.35, 0.7)	0.0058	0.6072
151.1118	208.7	Thymol	M+H	2	-0.02 (-0.26, 0.22)	0.35 (0.11, 0.59)	0.0322	0.7318
153.1023	25.9	2-Methoxy-(3 or 5 or 6)-isopropylpyrazine	M+H	2	0.33 (-0.16, 0.82)	-0.6 (-1.09, -0.11)	0.0107	0.6527
154.0587	84.6	Creatine	M+Na	1	-0.07 (-0.27, 0.13)	0.22 (0.03, 0.42)	0.0405	0.7347
166.0723	63.7	7-Methylguanine	M+H	2	0.32 (0.12, 0.51)	-0.09 (-0.28, 0.11)	0.0057	0.6072
166.0856	72.7	Phenylalanine	M+H	1	0.16 (0.07, 0.24)	0.01 (-0.08, 0.1)	0.0206	0.7283
166.1227	225.5	Pseudoephedrine	M+H	2	-0.61 (-1.27, 0.05)	0.52 (-0.14, 1.18)	0.0220	0.7318
169.9858	83.1	Taurine	M+2Na-H	1	0.31 (0.1, 0.52)	-0.12 (-0.33, 0.09)	0.0060	0.6072
170.1176	243.7	Homoarecoline	M+H	2	-0.32 (-0.65, 0)	0.16 (-0.16, 0.49)	0.0376	0.7347
171.0056	140	Glyceraldehyde 3-phosphate	M+H	2	-0.05 (-0.12, 0.02)	0.05 (-0.02, 0.12)	0.0486	0.7410
173.1173	216.8	Tetrahydrofurfuryl butyrate	M+H	2	0.67 (0.39, 0.95)	0.26 (-0.02, 0.54)	0.0409	0.7347
177.0546	23.5	Herniarin	M+H	2	0.08 (0.02, 0.13)	0.2 (0.14, 0.26)	0.0045	0.6072

177.0546	243	Herniarin	M+H	2	-0.23 (-0.44, -0.02)	0.09 (-0.12, 0.29)	0.0349	0.7347
181.1223	285.8	Tetramethyl-16-dioxaspironona-38-diene	M+H	2	-0.41 (-0.8, -0.03)	0.29 (-0.1, 0.68)	0.0134	0.6948
184.9856	227.6	Phosphohydroxypyruvic acid	M+H	2	0.33 (0.06, 0.59)	-0.09 (-0.36, 0.17)	0.0292	0.7318
185.1169	282.1	Nepetalic acid	M+H	2	-0.53 (-0.99, -0.06)	0.22 (-0.25, 0.69)	0.0288	0.7318
188.1281	119.1	N-Heptanoylglycine	M+H	2	-0.01 (-0.25, 0.23)	0.39 (0.15, 0.63)	0.0227	0.7318
191.1026	102.3	Diaminopimelic acid	M+H	2	-0.39 (-0.72, -0.07)	0.22 (-0.1, 0.54)	0.0096	0.6323
194.0805	15.6	Phenylacetylglycine	M+H	2	-0.37 (-0.98, 0.24)	0.59 (-0.02, 1.21)	0.0299	0.7318
198.1125	18.8	Metanephrine	M+H	2	-0.08 (-0.25, 0.08)	0.18 (0.02, 0.35)	0.0260	0.7318
199.039	289.1	3-Dechloroethylifosfamide	M+H	2	0.09 (-0.16, 0.35)	-0.31 (-0.57, -0.06)	0.0288	0.7318
203.1027	87.8	Alanyl-Hydroxyproline	M+H	2	-0.3 (-0.58, -0.02)	0.13 (-0.15, 0.41)	0.0323	0.7318
207.1743	267.7	delta-Methylionone	M+H	2	-0.61 (-1.18, -0.04)	0.24 (-0.33, 0.8)	0.0413	0.7347
209.0127	105.8	(Methylsulfinyl)-1-propenyl 2-propenyl disulfide	M+H	2	0.4 (0.1, 0.71)	-0.21 (-0.52, 0.09)	0.0058	0.6072
209.092	66.5	Kynurenine	M+H	1	0.01 (-0.09, 0.12)	-0.14 (-0.24, -0.03)	0.0442	0.7390
211.144	47.6	Cyclo(leucyl-prolyl)	M+H	2	0.23 (-0.26, 0.72)	-0.47 (-0.96, 0.02)	0.0498	0.7419
212.1182	28.1	Varenicline	M+H	2	0.2 (-1.18, 1.58)	-1.9 (-3.28, -0.52)	0.0372	0.7347
212.2009	13.4	Ethyl menthane carboxamide	M+H	2	-0.41 (-0.69, -0.14)	-0.01 (-0.29, 0.26)	0.0461	0.7390
213.9823	145.1	Chloroxine	M+H	2	0.23 (-0.17, 0.63)	-0.34 (-0.74, 0.05)	0.0455	0.7390
215.0702	26.4	Phenyl salicylate	M+H	2	-0.05 (-0.12, 0.02)	0.06 (-0.01, 0.13)	0.0251	0.7318
215.1277	252.3	5-Hexyltetrahydro-2-oxo-3-furancarboxylic acid	M+H	2	0.21 (-0.11, 0.53)	-0.36 (-0.68, -0.04)	0.0143	0.7012
217.1188	96.9	Prolyl-Threonine	M+H	2	-0.62 (-1.37, 0.14)	0.72 (-0.04, 1.47)	0.0159	0.7275
217.1797	27	12-Hydroxydodecanoic acid	M+H	2	0.14 (0.04, 0.23)	0.29 (0.2, 0.39)	0.0257	0.7318
221.1535	43.4	26-Di-tert-butylbenzoquinone	M+H	2	-0.25 (-0.46, -0.05)	0.19 (-0.02, 0.39)	0.0045	0.6072
224.128	12.2	Cerulenin	M+H	2	-0.09 (-0.3, 0.12)	0.23 (0.02, 0.44)	0.0333	0.7323
229.0856	229.4	Resveratrol	M+H	2	-0.46 (-0.84, -0.08)	0.11 (-0.27, 0.49)	0.0408	0.7347
233.0585	82.6	3-Chloro-1-(4-hydroxy-3-methoxyphenyl)-12-propanediol	M+H	2	-0.46 (-1.31, 0.39)	0.78 (-0.07, 1.63)	0.0449	0.7390
239.2003	18.5	Geranyl 3-methylbutanoate	M+H	2	-0.17 (-0.34, 0)	0.12 (-0.05, 0.29)	0.0183	0.7283
245.0799	87.2	3345-Tetrahydroxystilbene	M+H	2	0.39 (0.16, 0.62)	-0.15 (-0.39, 0.08)	0.0022	0.5656
245.1859	54.2	Isoleucyl-Isoleucine	M+H	2	0.45 (0.21, 0.69)	0.02 (-0.23, 0.26)	0.0139	0.6948
251.1276	234.7	Ubiquinone-1	M+H	2	-0.08 (-0.38, 0.22)	0.4 (0.1, 0.7)	0.0293	0.7318
251.2003	228.2	Norambreinolide	M+H	2	0 (-0.25, 0.25)	0.45 (0.2, 0.7)	0.0144	0.7012
255.1952	19.5	Lubiminol	M+H	2	0.12 (-0.01, 0.25)	0.33 (0.2, 0.46)	0.0294	0.7318
256.2633	150.7	Palmitic amide	M+H	2	0.14 (-0.11, 0.38)	-0.27 (-0.52, -0.03)	0.0212	0.7283
261.1805	80.8	Carisoprodol	M+H	2	0.93 (-0.42, 2.27)	-1.14 (-2.48, 0.2)	0.0339	0.7347
269.2261	43.7	Vitamin A	M+H-H2O	1	0.06 (-0.04, 0.15)	-0.1 (-0.19, -0.01)	0.0196	0.7283
279.2314	41.9	Linolenic acid	M+H	2	0.11 (-0.18, 0.4)	-0.33 (-0.62, -0.05)	0.0335	0.7323
281.1162	51.4	Levosimendan	M+H	2	0.04 (-0.62, 0.7)	-1.74 (-2.4, -1.08)	0.0005	0.5624
284.0091	87.9	Risedronate	M+H	2	-0.1 (-0.3, 0.11)	0.42 (0.22, 0.63)	0.0012	0.5624
297.242	42.5	13S-hydroxyoctadecadienoic acid	M+H	2	0.19 (-0.12, 0.5)	-0.28 (-0.59, 0.03)	0.0355	0.7347
299.1259	24	7C-aglycone	M+H	2	-0.2 (-0.4, -0.01)	0.15 (-0.04, 0.35)	0.0134	0.6948
313.1428	265.7	4-(3-Methyl-1-butenyl)-3345-tetrahydroxystilbene	M+H	2	0.43 (-0.16, 1.02)	-0.44 (-1.03, 0.15)	0.0426	0.7347
315.3275	16.9	120-Eicosanediol	M+H	2	0 (-0.41, 0.41)	0.79 (0.38, 1.2)	0.0086	0.6323

351.0989	15.4	Penicillin V	M+H	2	-0.11 (-0.24, 0.02)	0.08 (-0.05, 0.21)	0.0423	0.7347
355.1095	105.6	Flumioxazin	M+H	2	0.37 (-0.01, 0.74)	-0.17 (-0.55, 0.2)	0.0450	0.7390
365.105	103.7	Disaccharide	M+Na	1	0.57 (-0.13, 1.28)	-0.53 (-1.23, 0.18)	0.0323	0.7318
389.2508	24.6	56-Dihydroxyprostaglandin F1a	M+H	2	-0.37 (-0.76, 0.03)	0.39 (0, 0.78)	0.0093	0.6323
399.1221	25	Methyl acrylate-divinylbenzene completely hydrolyzed copolymer	M+H	2	-0.1 (-0.16, -0.03)	0.07 (0.01, 0.14)	0.0006	0.5624
428.3732	45.1	Stearoylcarnitine	M+H	2	0.04 (-0.15, 0.23)	-0.25 (-0.44, -0.06)	0.0338	0.7347
433.9982	83	Quinoline yellow	M+H	2	-0.13 (-0.26, -0.01)	0.08 (-0.04, 0.21)	0.0206	0.7283
459.239	41.4	3-Sulfodeoxycholic acid	M+H	2	-0.18 (-0.42, 0.07)	0.2 (-0.04, 0.45)	0.0315	0.7318
476.2769	54.6	LysoPE(18:3)	M+H	2	0.02 (-0.6, 0.64)	-1.04 (-1.66, -0.42)	0.0217	0.7318
524.3714	57.9	LysoPC(18:0)	M+H	1	-0.03 (-0.15, 0.1)	-0.21 (-0.34, -0.09)	0.0403	0.7347
528.3077	53.8	LysoPE(22:5)	M+H	2	-0.15 (-0.57, 0.27)	-0.79 (-1.21, -0.37)	0.0353	0.7347
546.3552	58.4	LysoPC(20:3)	M+H	2	0.02 (-0.11, 0.16)	-0.19 (-0.32, -0.05)	0.0339	0.7347
548.3717	57.5	LysoPC(20:2)	M+H	2	0.2 (0, 0.39)	-0.1 (-0.29, 0.1)	0.0397	0.7347
556.2786	63.6	Enkephalin L	M+H	2	0.07 (-0.52, 0.66)	-0.83 (-1.42, -0.25)	0.0364	0.7347
557.4561	42.7	DG(33:4)	M+H-H2O	2	-0.03 (-0.45, 0.39)	-0.7 (-1.11, -0.28)	0.0296	0.7318
570.3533	57.3	LysoPC(22:5)	M+H	2	-0.03 (-0.16, 0.09)	-0.24 (-0.36, -0.11)	0.0257	0.7318
572.3694	57.3	LysoPC(22:4)	M+H	2	0.14 (-0.07, 0.34)	-0.24 (-0.44, -0.04)	0.0113	0.6570
577.4823	42.8	DG(33:3)	M+H	2	0.05 (-0.32, 0.42)	-0.51 (-0.87, -0.14)	0.0372	0.7347
611.3116	62.5	Isoliensinine	M+H	2	0.29 (-0.07, 0.65)	-0.23 (-0.59, 0.12)	0.0429	0.7356
647.5124	55.7	SM(d18:112:0)	M+H	2	0.31 (-0.1, 0.72)	-0.46 (-0.87, -0.05)	0.0107	0.6527
730.5381	50.4	PC(32:2)	M+H	2	0.15 (-0.01, 0.32)	-0.18 (-0.34, -0.01)	0.0065	0.6072
732.5535	50.3	PC(32:1)	M+H	2	0.07 (-0.11, 0.24)	-0.24 (-0.41, -0.06)	0.0186	0.7283
756.5513	49.7	PC(34:3)	M+H	2	0.16 (0.05, 0.26)	-0.06 (-0.16, 0.05)	0.0079	0.6323
772.5838	49.7	PC(35:2)	M+H	2	0.08 (-0.02, 0.18)	0.29 (0.19, 0.39)	0.0044	0.6072
797.5274	63.2	PG(38:5)	M+H	2	-0.07 (-0.43, 0.29)	-0.64 (-1, -0.27)	0.0317	0.7318
812.6128	48.9	PC(38:3)	M+H	2	0.05 (-0.05, 0.14)	-0.1 (-0.19, 0)	0.0403	0.7347
820.5825	48.8	PE(44:6)	M+H	2	0.14 (-0.22, 0.51)	0.72 (0.35, 1.08)	0.0309	0.7318
824.6504	48.8	PC(40:3)	M+H	2	-0.25 (-0.55, 0.04)	0.35 (0.05, 0.64)	0.0061	0.6072
830.5652	50.2	PC(40:8)	M+H	2	0.24 (0.07, 0.41)	0 (-0.17, 0.17)	0.0487	0.7410
838.6271	49.4	PC(40:4)	M+H	2	0.03 (-0.09, 0.16)	-0.19 (-0.31, -0.06)	0.0186	0.7283
976.3683	73	5-Methyltetrahydropteroylpentaglutamate	M+H	2	-0.18 (-0.43, 0.07)	0.21 (-0.04, 0.46)	0.0310	0.7318
**B) C18 / -ESI column**								
**m/z**	**Time (s)**	**Compound Name**	**Adduct**	**MSI Level**	**Control Group Mean Δ (95% CI)**	**Treatment Group Mean Δ (95% CI)**	**p-value[a]**	**q-value[b]**
103.04	38.9	3-Hydroxybutyric acid	M-H	1	-0.05 (-0.23, 0.14)	0.35 (0.16, 0.54)	0.0051	0.8785
103.0593	16.7	1-Pentanethiol	M-H	2	-0.31 (-0.93, 0.32)	0.81 (0.18, 1.43)	0.0152	0.8969
104.0353	40.2	Serine	M-H	2	-0.13 (-0.26, 0)	0.2 (0.07, 0.33)	0.0009	0.7258
116.0353	34.8	Acetylglycine	M-H	2	-0.11 (-0.41, 0.2)	0.4 (0.09, 0.71)	0.0241	0.9366
124.0074	44.1	Taurine	M-H	2	0.27 (0.03, 0.52)	-0.13 (-0.38, 0.11)	0.0222	0.9366
138.0197	11.9	Hydroxynicotinic acid	M-H	2	-0.05 (-0.14, 0.03)	0.11 (0.03, 0.2)	0.0080	0.8785
139.0168	40.4	3-Hydroxybutyric acid	M+Cl	1	0 (-0.24, 0.24)	0.36 (0.12, 0.6)	0.0371	0.9396
146.0823	35.7	2-Amino-4-hydroxy-3-methylpentanoic acid	M-H	2	-0.32 (-0.55, -0.09)	0.07 (-0.16, 0.3)	0.0238	0.9366
147.0663	18.4	Mevalonic acid	M-H	1	-0.14 (-0.49, 0.21)	0.38 (0.03, 0.73)	0.0436	0.9657

152.0354	294.3	3-Hydroxyanthranilic acid	M-H	2	-0.41 (-0.74, -0.09)	0.1 (-0.23, 0.42)	0.0307	0.9366
155.0099	16.2	Orotic acid	M-H	2	-0.16 (-0.28, -0.04)	0.09 (-0.03, 0.21)	0.0055	0.8785
157.0507	290.5	Succinylacetone	M-H	2	0.38 (0.15, 0.62)	0.04 (-0.19, 0.28)	0.0452	0.9657
160.0616	36.5	Aminoadipic acid	M-H	2	-0.16 (-0.32, -0.01)	0.06 (-0.09, 0.22)	0.0456	0.9676
172.0616	291.1	Acetylglutamate 5-semialdehyde	M-H	2	-0.1 (-0.29, 0.09)	0.28 (0.09, 0.47)	0.0074	0.8785
174.0561	46.5	Indole-3-acetic acid	M-H	1	-0.07 (-0.31, 0.16)	0.27 (0.03, 0.5)	0.0467	0.9676
181.018	52.1	Dipropyl trisulfide	M-H	2	-0.04 (-0.07, 0)	0.02 (-0.02, 0.06)	0.0339	0.9366
189.0406	41.7	3-Dehydroquinate	M-H	2	0.04 (-0.08, 0.16)	-0.19 (-0.31, -0.07)	0.0098	0.8785
217.1081	289.4	3-Hydroxysebacic acid	M-H	2	0.03 (-0.13, 0.19)	0.27 (0.1, 0.43)	0.0499	0.9676
228.0409	273.2	Amiloride	M-H	2	0.37 (0.18, 0.56)	0.1 (-0.09, 0.28)	0.0410	0.9518
229.1081	10.3	Dicyclohexyl disulfide	M-H	2	-0.25 (-0.59, 0.08)	0.3 (-0.04, 0.63)	0.0234	0.9366
237.186	199	Geranyl 3-methylbutanoate	M-H	2	0.14 (-0.19, 0.46)	-0.38 (-0.71, -0.06)	0.0306	0.9366
243.1237	148.1	Polyethylene oxidized	M-H	2	-0.54 (-0.99, -0.09)	0.47 (0.02, 0.92)	0.0030	0.8785
252.0465	47.8	Sulfamethoxazole	M-H	2	-0.01 (-0.11, 0.09)	0.16 (0.06, 0.27)	0.0164	0.9278
275.2015	212.3	19-Norandrosterone	M-H	2	0.21 (0.02, 0.41)	-0.15 (-0.35, 0.05)	0.0125	0.8785
277.2173	226.8	Linolenic acid	M-H	1	0.26 (0.09, 0.43)	-0.09 (-0.26, 0.08)	0.0057	0.8785
311.1352	187	Olanzapine	M-H	2	-0.05 (-0.11, 0.02)	0.05 (-0.02, 0.12)	0.0477	0.9676
323.2228	225.2	1-Acetoxy-2-hydroxy-16-heptadecyn-4-one	M-H	2	0.25 (0.05, 0.45)	-0.15 (-0.35, 0.05)	0.0076	0.8785
349.2379	244.1	Tetrahydrocorticosterone	M-H	2	0.18 (-0.1, 0.46)	-0.29 (-0.57, -0.01)	0.0236	0.9366
357.28	266.2	Tetracosapentaenoic acid (24:5n-6)	M-H	2	0.18 (0.03, 0.33)	-0.13 (-0.28, 0.02)	0.0072	0.8785
409.2725	210.8	58-Epoxy-58-dihydro-10-apo-by-carotene-310-diol	M-H	2	0.05 (-0.34, 0.43)	0.62 (0.24, 1.01)	0.0392	0.9518
435.2754	86.4	simvastatin hydroxy acid	M-H	2	-0.61 (-1.1, -0.12)	0.16 (-0.33, 0.65)	0.0311	0.9366
435.2778	278.9	Simvastatin hydroxy acid	M-H	2	-0.34 (-0.93, 0.25)	0.52 (-0.07, 1.12)	0.0449	0.9657
445.3324	240.8	13-Carboxy-gamma-tocopherol	M-H	2	0.12 (-0.08, 0.32)	-0.29 (-0.49, -0.09)	0.0064	0.8785
450.2612	196	LysoPE(16:1)	M-H	2	-0.09 (-0.35, 0.17)	-0.51 (-0.76, -0.25)	0.0269	0.9366
473.3625	256.9	Soyasapogenol A	M-H	2	0.05 (-0.13, 0.22)	-0.32 (-0.49, -0.15)	0.0043	0.8785
474.2625	193.3	LysoPE(18:3)	M-H	2	0 (-0.32, 0.32)	-0.48 (-0.8, -0.16)	0.0422	0.9614
484.2644	177.5	Sarcodon scabrosus Depsipeptide	M-H	2	0.26 (-0.1, 0.62)	-0.24 (-0.6, 0.11)	0.0493	0.9676
497.2864	183.6	Polyporusterone B	M+Na-2H	2	0.01 (-0.42, 0.43)	-0.6 (-1.02, -0.18)	0.0469	0.9676
510.2835	183.9	Cytochalasin Ppho	M-H	2	0.23 (-0.29, 0.75)	-0.63 (-1.15, -0.11)	0.0239	0.9366
526.2937	208	LysoPE(22:5)	M-H	2	-0.13 (-0.26, 0.01)	-0.35 (-0.48, -0.21)	0.0247	0.9366
528.3091	216.9	LysoPE(22:4)	M-H	2	-0.08 (-0.25, 0.09)	-0.36 (-0.53, -0.19)	0.0240	0.9366
569.2722	166	Ganoderic acid F	M-H	2	0.07 (-0.17, 0.32)	-0.44 (-0.68, -0.2)	0.0043	0.8785
570.2781	183.9	Kinetensin 4-7	M-H	2	0.27 (-0.29, 0.82)	-0.6 (-1.16, -0.04)	0.0324	0.9366
573.4528	276.8	DG(33:4)	M-H	2	0.02 (-0.4, 0.44)	-0.63 (-1.05, -0.2)	0.0354	0.9396
577.4837	293.8	DG(33:2)	M-H	2	0.09 (-0.38, 0.56)	-0.69 (-1.16, -0.22)	0.0242	0.9366
579.3054	209.7	Hordatine B	M-H	2	-0.27 (-0.67, 0.14)	0.37 (-0.04, 0.77)	0.0309	0.9366
591.3905	211.7	Tuberoside	M-H	2	0.25 (-0.04, 0.53)	-0.18 (-0.46, 0.1)	0.0452	0.9657
682.303	248	Chitin	M-H	2	-0.18 (-0.37, 0.01)	0.14 (-0.05, 0.33)	0.0236	0.9366
790.5627	265.9	PS(36:0)	M-H	2	0.45 (-0.1, 1)	-0.54 (-1.09, 0.01)	0.0157	0.8982

[a] Mean change values, 95% CIs, and p-values were calculated from linear regression models adjusted for baseline values for each metabolite. Only showing results for m/z features that were confirmed (Level 1) or putatively annotated (Level 2) according to Metabolomics Standard Initiative (MSI) critiera.
[b] q-values were calculated using the Benjamini-Hochberg method

Table S3: Concentrations for quantified and confirmed metabolites that were differentially changed from baseline to week 8 in the diet treatment group compared to control group

		Control Group (n=20)		Diet Group (n=20)				
Metabolite[a]	Adduct	Week 0 Mean (SD)	Week 8 Mean (SD)	Week 0 Mean (SD)	Week 8 Mean (SD)	HMDB Range (µM)	HMDB Ref Population	Source/ Link
Serine	M+H	100.1 (15.3)	95.6 (20.5)	105.7 (22.7)	111.3 (17.1)	60.0-180.0	Adult (> 18 yrs)	Molecular You
Acetylglycine	M+H	0.04 (0.02)	0.04 (0.02)	0.04 (0.02)	0.05 (0.03)	109.4 ± 85.6	Adult (> 18 yrs)	21359215
2-Aminobutyric acid	M+H	8 (2)	7.9 (1.8)	8.3 (1.6)	9.7 (2.1)	15.0-31.0	Adult (> 18 yrs)	12297216
Creatine	M+H	72.1 (25.2)	68.4 (22.2)	70.7 (24.1)	79.9 (20.1)	87 ± 19	Adult (>18 yrs)	15024124
Kynurenine	M+H	2.2 (0.5)	2.2 (0.5)	2.2 (0.4)	2 (0.4)	0.7-3.0	Adult (> 18 yrs)	8634758
Indole-3-acetatic acid	M-H	1.9 (1.8)	1.7 (1.1)	1.2 (0.5)	1.5 (0.7)	2.9 ± 1.7	Adult (> 18 yrs)	22626821
3-Hydroxybutyric acid	M-H	220.1 (83.2)	226.2 (75)	251.9 (62.6)	300.7 (104.3)	10.6-143.2	Adult (> 18 yrs)	21359215
Linolenic acid	M-H	77.5 (33.2)	92.6 (35.0)	82.6 (30.3)	74.8 (22.1)	46.1 ± 21.7	Adult (> 18 yrs)	27329611
Retinol (Vitamin A)	M+H-H2O	7.6 (1.7)	7.8 (1.8)	7.6 (1.7)	7.1 (1.7)	0.3-2.1	Adolescent (13-18 yrs)	7657478

[a] Only showing data for select confirmed metabolites that were quantified using reference standardization (Go, 2015; Liu, 2020) and were differentially changed from baseline to week 8 in the diet treatment group compared to control group, as shown in the main text in Figure 3.

Table S4: Least squares (LS) mean change values and 95% confidence intervals (CIs) by treatment group for the first and second multidimensional scaling (MDS) axis from PCoA analysis based on Bray-Curtis dissimilarity.

			Control Group (n=8)		Diet Treatment Group (n=10)			
**Taxa Level**	**Axis**	**MDS Mean**	**Mean Δ[a]**	**(95% CI)**	**Mean Δ[a]**	**(95% CI)**	**p-value[a]**	**q-value[b]**
Phylum	MDS1	1.59E-17	0.141	(0.01, 0.27)	0.005	(-0.11, 0.12)	0.147	0.552
Phylum	MDS2	2.43E-17	-0.057	(-0.17, 0.06)	0.020	(-0.08, 0.12)	0.308	0.633
Class	MDS1	-1.85E-17	0.000	(-0.11, 0.11)	-0.095	(-0.2, 0.01)	0.196	0.489
Class	MDS2	6.07E-17	-0.086	(-0.23, 0.06)	-0.007	(-0.14, 0.13)	0.417	0.606
Order	MDS1	-1.06E-17	-0.035	(-0.15, 0.08)	-0.099	(-0.2, 0.01)	0.393	0.607
Order	MDS2	-2.47E-17	-0.147	(-0.29, 0.01)	-0.034	(-0.16, 0.1)	0.236	0.540
Family	MDS1	1.57E-17	0.019	(-0.07, 0.11)	-0.043	(-0.13, 0.04)	0.314	0.678
Family	MDS2	1.39E-17	0.004	(-0.08, 0.09)	-0.012	(-0.09, 0.07)	0.780	0.821
Genus	MDS1	2.65E-17	-0.012	(-0.07, 0.04)	0.027	(-0.02, 0.08)	0.286	0.859
Genus	MDS2	-1.32E-17	0.034	(-0.06, 0.13)	-0.002	(-0.09, 0.08)	0.561	0.885
OTU	MDS1	4.80E-17	-0.008	(-0.06, 0.04)	-0.005	(-0.05, 0.04)	0.922	0.922
OTU	MDS2	8.87E-18	-0.030	(-0.09, 0.03)	-0.024	(-0.08, 0.03)	0.875	0.922

[a] Mean change values, 95% CIs, and p-values were calculated from linear regression models adjusted for baseline values for each metabolite. Only showing results for the first and second multidimensional scaling (MDS) axes.

[b] q-values were calculated using the Benjamini-Hochberg method

Table S5: Least squares (LS) mean change values and 95% confidence intervals (CIs) by treatment group for rarefied microbial diversity measures

	Control Group (n=8)		Diet treatment group (n=10)			
	**Mean Δ[a]**	**(95% CI)**	**Mean Δ[a]**	**(95% CI)**	**p-value[a]**	**q-value[b]**
**Taxa**			**Shannon Index**			
Phylum	0.073	(-0.02, 0.17)	-0.034	(-0.12, 0.05)	0.111	0.400
Class	0.074	(-0.01, 0.16)	0.014	(-0.06, 0.09)	0.317	0.400
Order	0.076	(-0.01, 0.16)	0.021	(-0.05, 0.09)	0.359	0.400
Family	0.127	(0, 0.25)	0.004	(-0.11, 0.12)	0.177	0.400
Genus	0.100	(0.03, 0.17)	0.056	(-0.01, 0.12)	0.366	0.400
OTU	0.102	(0, 0.2)	0.041	(-0.05, 0.13)	0.355	0.400
**Taxa**			**Inverse Simpson Index**			
Phylum	0.076	(-0.08, 0.23)	-0.076	(-0.21, 0.06)	0.159	0.349
Class	0.068	(-0.09, 0.22)	-0.047	(-0.18, 0.09)	0.288	0.349
Order	0.069	(-0.09, 0.23)	-0.043	(-0.18, 0.1)	0.305	0.349
Family	0.449	(-0.13, 1.03)	-0.214	(-0.72, 0.3)	0.115	0.349
Genus	0.772	(-0.68, 2.22)	0.518	(-0.78, 1.81)	0.785	0.822
OTU	2.451	(-0.31, 5.21)	0.420	(-2.05, 2.89)	0.261	0.349
**Taxa**			**Richness**			
Phylum	0.778	(0.16, 1.4)	-0.302	(-0.85, 0.24)	**0.018**	0.113
Class	1.844	(0.66, 3.03)	0.434	(-0.62, 1.49)	**0.090**	0.271
Order	2.098	(0.65, 3.55)	0.821	(-0.46, 2.11)	0.196	0.395
Family	2.725	(0.79, 4.66)	1.310	(-0.42, 3.04)	0.268	0.398
Genus	3.292	(0.98, 5.61)	1.936	(-0.13, 4)	0.367	0.435
OTU	3.981	(0.73, 7.23)	2.525	(-0.38, 5.43)	0.488	0.464
**Taxa**			**Evenness**			
Phylum	-0.023	(-0.09, 0.04)	0.008	(-0.05, 0.06)	0.444	0.691
Class	-0.030	(-0.07, 0.01)	0.006	(-0.03, 0.04)	0.223	0.691
Order	-0.033	(-0.08, 0.01)	0.002	(-0.04, 0.04)	0.262	0.691
Family	0.001	(-0.03, 0.04)	-0.006	(-0.04, 0.02)	0.756	0.831
Genus	0.005	(-0.01, 0.02)	0.003	(-0.01, 0.02)	0.859	0.920
OTU	0.008	(-0.01, 0.03)	-0.002	(-0.02, 0.02)	0.454	0.691

[a] Mean change values, 95% confidence intervals, and p-values were calculated as the least squares means from linear regression models adjusted for baseline values. Bold values indicate a raw p-value that was stastistically significant (p<0.05) or borderline significant (p<0.10).
[b] q-values were calculated using the Benjamini-Hochberg method

Table S6: Mean change values and 95% confidence intervals (CIs) by treatment group for the log-normalized relative abundance of bacteria according to each taxonomic level

		Control Group (n=10)		Diet Group (n=8)			
**Taxa**	**Name**	**Mean Δ[a]**	**(95% CI)**	**Mean Δ[a]**	**(95% CI)**	**p-value[a]**	**q-value[b]**
Phylum	P_Firmicutes	0.015	(-0.01, 0.04)	-0.015	(-0.05, 0.02)	0.183	0.730
	P_Proteobacteria	0.166	(-0.3, 0.63)	0.425	(-0.11, 0.96)	0.491	0.741
	P_Bacteroidetes	-0.041	(-0.17, 0.08)	0.015	(-0.13, 0.16)	0.556	0.741
	P_Actinobacteria	0.159	(-0.15, 0.47)	0.222	(-0.13, 0.57)	0.787	0.787
Class	P_Firmicutes; C_Clostridia	0.011	(-0.02, 0.04)	-0.015	(-0.05, 0.02)	0.214	0.249
	P_Proteobacteria; C_Betaproteobacteria	0.093	(-0.41, 0.6)	0.485	(-0.09, 1.06)	0.332	0.226
	P_Bacteroidetes; C_Bacteroidia	-0.041	(-0.17, 0.08)	0.015	(-0.13, 0.16)	0.556	0.619
	P_Actinobacteria; C_Coriobacteriia	0.361	(-0.02, 0.74)	0.202	(-0.22, 0.62)	0.559	0.833
	P_Firmicutes; C_Bacilli	0.430	(0.05, 0.81)	0.271	(-0.15, 0.7)	0.564	0.833
	P_Actinobacteria; C_Actinobacteria	0.013	(-0.5, 0.53)	0.163	(-0.42, 0.75)	0.699	0.527
	P_Proteobacteria; C_Gammaproteobacteria	0.335	(-0.27, 0.95)	0.473	(-0.21, 1.16)	0.754	0.833
	P_Proteobacteria; C_Deltaproteobacteria	0.283	(-0.18, 0.75)	0.214	(-0.31, 0.74)	0.839	0.833
	P_Firmicutes; C_Erysipelotrichi	0.117	(-0.26, 0.49)	0.168	(-0.25, 0.59)	0.851	0.833
Order	P_Firmicutes; C_Clostridia; O_Clostridiales	0.011	(-0.02, 0.04)	-0.015	(-0.05, 0.02)	0.214	0.249
	P_Proteobacteria; C_Betaproteobacteria; O_Burkholderiales	0.093	(-0.41, 0.6)	0.485	(-0.09, 1.06)	0.332	0.226
	P_Proteobacteria; C_Gammaproteobacteria; O_Enterobacteriales	0.249	(-0.4, 0.9)	0.640	(-0.09, 1.37)	0.408	0.694
	P_Firmicutes; C_Bacilli; O_Lactobacillales	0.533	(0.11, 0.96)	0.283	(-0.19, 0.76)	0.417	0.694
	P_Bacteroidetes; C_Bacteroidia; O_Bacteroidales	-0.041	(-0.17, 0.08)	0.015	(-0.13, 0.16)	0.556	0.619
	P_Actinobacteria; C_Coriobacteriia; O_Coriobacteriales	0.361	(-0.02, 0.74)	0.202	(-0.22, 0.62)	0.559	0.694
	P_Actinobacteria; C_Actinobacteria; O_Bifidobacteriales	0.013	(-0.5, 0.53)	0.163	(-0.42, 0.75)	0.699	0.527
	P_Proteobacteria; C_Deltaproteobacteria; O_Desulfovibrionales	0.283	(-0.18, 0.75)	0.214	(-0.31, 0.74)	0.839	0.793
	P_Firmicutes; C_Erysipelotrichi; O_Erysipelotrichales	0.117	(-0.26, 0.49)	0.168	(-0.25, 0.59)	0.851	0.694
Family	P_Firmicutes; C_Bacilli; O_Lactobacillales; F_Lactobacillaceae	0.229	(-0.08, 0.54)	-0.225	(-0.58, 0.13)	**0.060**	0.779
	P_Firmicutes; C_Clostridia; O_Clostridiales; F_Lachnospiraceae	0.018	(-0.04, 0.07)	-0.060	(-0.12, 0)	**0.089**	0.779
	P_Bacteroidetes; C_Bacteroidia; O_Bacteroidales; F_Prevotellaceae	0.079	(-0.19, 0.35)	-0.249	(-0.56, 0.06)	0.127	0.779
	P_Bacteroidetes; C_Bacteroidia; O_Bacteroidales; F_Bacteroidaceae	-0.082	(-0.2, 0.04)	0.047	(-0.09, 0.18)	0.156	0.779
	P_Firmicutes; C_Clostridia; O_Clostridiales; F_	-0.132	(-0.52, 0.25)	0.241	(-0.19, 0.67)	0.191	0.779

	P_Firmicutes; C_Clostridia; O_Clostridiales; F_Clostridiaceae	-0.130	(-0.36, 0.1)	0.091	(-0.17, 0.35)	0.212	0.779
	P_Firmicutes; C_Clostridia; O_Clostridiales; F_Mogibacteriaceae	0.313	(-0.04, 0.67)	0.641	(0.24, 1.04)	0.235	0.779
	P_Bacteroidetes; C_Bacteroidia; O_Bacteroidales; F_Rikenellaceae	0.025	(-0.34, 0.39)	0.330	(-0.08, 0.74)	0.260	0.779
	P_Firmicutes; C_Clostridia; O_Clostridiales; F_Christensenellaceae	-0.109	(-0.62, 0.41)	0.287	(-0.29, 0.86)	0.293	0.782
	P_Proteobacteria; C_Betaproteobacteria; O_Burkholderiales; F_Alcaligenaceae	0.093	(-0.41, 0.6)	0.485	(-0.09, 1.06)	0.332	0.797
	P_Proteobacteria; C_Gammaproteobacteria; O_Enterobacteriales; F_Enterobacteriaceae	0.249	(-0.4, 0.9)	0.640	(-0.09, 1.37)	0.408	0.823
	P_Firmicutes; C_Clostridia; O_Clostridiales; F_Ruminococcaceae	0.175	(0.11, 0.24)	0.211	(0.14, 0.28)	0.438	0.823
	P_Bacteroidetes; C_Bacteroidia; O_Bacteroidales; F_Paraprevotellaceae	0.172	(-0.15, 0.49)	-0.007	(-0.37, 0.35)	0.446	0.823
	P_Firmicutes; C_Bacilli; O_Lactobacillales; F_Enterococcaceae	0.209	(-0.41, 0.83)	0.486	(-0.21, 1.18)	0.534	0.838
	P_Firmicutes; C_Bacilli; O_Lactobacillales; F_Streptococcaceae	0.370	(-0.21, 0.95)	0.109	(-0.54, 0.76)	0.535	0.838
	P_Actinobacteria; C_Coriobacteriia; O_Coriobacteriales; F_Coriobacteriaceae	0.361	(-0.02, 0.74)	0.202	(-0.22, 0.62)	0.559	0.838
	P_Bacteroidetes; C_Bacteroidia; O_Bacteroidales; F_Porphyromonadaceae	-0.190	(-0.77, 0.39)	0.016	(-0.63, 0.66)	0.628	0.887
	P_Actinobacteria; C_Actinobacteria; O_Bifidobacteriales; F_Bifidobacteriaceae	0.013	(-0.5, 0.53)	0.163	(-0.42, 0.75)	0.699	0.932
	P_Bacteroidetes; C_Bacteroidia; O_Bacteroidales; F_Barnesiellaceae	-0.097	(-0.4, 0.21)	-0.036	(-0.38, 0.31)	0.787	0.932
	P_Proteobacteria; C_Deltaproteobacteria; O_Desulfovibrionales; F_Desulfovibrionaceae	0.283	(-0.18, 0.75)	0.214	(-0.31, 0.74)	0.839	0.932
	P_Firmicutes; C_Erysipelotrichi; O_Erysipelotrichales; F_Erysipelotrichaceae	0.117	(-0.26, 0.49)	0.168	(-0.25, 0.59)	0.851	0.932
	P_Firmicutes; C_Clostridia; O_Clostridiales; F__Unclassified	0.074	(-0.17, 0.32)	0.042	(-0.23, 0.31)	0.855	0.932
	P_Firmicutes; C_Clostridia; O_Clostridiales; F_Veillonellaceae	-0.034	(-0.49, 0.42)	-0.013	(-0.53, 0.5)	0.949	0.990
	P_Bacteroidetes; C_Bacteroidia; O_Bacteroidales; F_Odoribacteraceae	0.130	(-0.3, 0.56)	0.129	(-0.36, 0.61)	0.996	0.996
Genus	P_Firmicutes; C_Clostridia; O_Clostridiales; F_Ruminococcaceae; G__Unclassified	-0.083	(-0.26, 0.1)	0.327	(0.13, 0.53)	**0.006**	0.238
	P_Firmicutes; C_Clostridia; O_Clostridiales; F_Veillonellaceae; G_Phascolarctobacterium	-0.211	(-0.49, 0.07)	0.250	(-0.06, 0.56)	**0.035**	0.702
	P_Firmicutes; C_Bacilli; O_Lactobacillales; F_Lactobacillaceae; G_Lactobacillus	0.229	(-0.08, 0.54)	-0.225	(-0.58, 0.13)	**0.060**	0.702
	P_Firmicutes; C_Erysipelotrichi; O_Erysipelotrichales; F_Erysipelotrichaceae; G_Eubacterium	0.262	(-0.06, 0.59)	-0.150	(-0.51, 0.21)	**0.096**	0.702
	P_Firmicutes; C_Clostridia; O_Clostridiales; F_Lachnospiraceae; G_Lachnobacterium	0.461	(-0.15, 1.07)	-0.313	(-1, 0.37)	**0.097**	0.702

P_Bacteroidetes; C_Bacteroidia; O_Bacteroidales; F_Prevotellaceae; G_Prevotella	0.079	(-0.19, 0.35)	-0.249	(-0.56, 0.06)	0.127	0.702
P_Firmicutes; C_Clostridia; O_Clostridiales; F_Veillonellaceae; G_Dialister	0.164	(-0.21, 0.54)	-0.236	(-0.65, 0.18)	0.149	0.702
P_Bacteroidetes; C_Bacteroidia; O_Bacteroidales; F_Bacteroidaceae; G_Bacteroides	-0.082	(-0.2, 0.04)	0.047	(-0.09, 0.18)	0.156	0.702
P_Proteobacteria; C_Gammaproteobacteria; O_Enterobacteriales; F_Enterobacteriaceae; G_Escherichia	0.096	(-0.47, 0.67)	0.703	(0.06, 1.34)	0.161	0.702
P_Firmicutes; C_Erysipelotrichi; O_Erysipelotrichales; F_Erysipelotrichaceae; G_Clostridium	-0.005	(-0.28, 0.27)	0.286	(-0.02, 0.59)	0.167	0.702
P_Firmicutes; C_Clostridia; O_Clostridiales; F_Unclassified; G_Unclassified	-0.132	(-0.52, 0.25)	0.241	(-0.19, 0.67)	0.191	0.702
P_Firmicutes; C_Clostridia; O_Clostridiales; F_Lachnospiraceae; G_Ruminococcus	-0.007	(-0.14, 0.12)	-0.131	(-0.28, 0.02)	0.201	0.702
P_Firmicutes; C_Clostridia; O_Clostridiales; F_Mogibacteriaceae; G_Unclassified	0.313	(-0.04, 0.67)	0.641	(0.24, 1.04)	0.235	0.760
P_Bacteroidetes; C_Bacteroidia; O_Bacteroidales; F_Rikenellaceae; G_Unclassified	0.029	(-0.33, 0.39)	0.332	(-0.08, 0.74)	0.261	0.762
P_Firmicutes; C_Clostridia; O_Clostridiales; F_Ruminococcaceae; G_Gemmiger	0.006	(-0.08, 0.09)	0.076	(-0.02, 0.17)	0.272	0.762
P_Firmicutes; C_Clostridia; O_Clostridiales; F_Christensenellaceae; G_Unclassified	-0.109	(-0.62, 0.41)	0.287	(-0.29, 0.86)	0.293	0.766
P_Firmicutes; C_Clostridia; O_Clostridiales; F_Lachnospiraceae; G_Roseburia	0.180	(0.04, 0.32)	0.076	(-0.08, 0.23)	0.310	0.766
P_Proteobacteria; C_Betaproteobacteria; O_Burkholderiales; F_Alcaligenaceae; G_Sutterella	0.093	(-0.41, 0.6)	0.485	(-0.09, 1.06)	0.332	0.775
P_Bacteroidetes; C_Bacteroidia; O_Bacteroidales; F_Paraprevotellaceae; G_Paraprevotella	0.222	(-0.09, 0.53)	0.021	(-0.33, 0.37)	0.373	0.814
P_Firmicutes; C_Clostridia; O_Clostridiales; F_Lachnospiraceae; G_Unclassified	0.187	(0.09, 0.29)	0.125	(0.01, 0.24)	0.391	0.814
P_Firmicutes; C_Clostridia; O_Clostridiales; F_Ruminococcaceae; G_Ruminococcus	-0.071	(-0.26, 0.12)	0.041	(-0.17, 0.26)	0.425	0.814
P_Firmicutes; C_Clostridia; O_Clostridiales; F_Clostridiaceae; G_SMB53	0.462	(-0.16, 1.09)	0.139	(-0.56, 0.84)	0.477	0.814
P_Firmicutes; C_Clostridia; O_Clostridiales; F_Lachnospiraceae; G_Anaerostipes	-0.075	(-0.72, 0.57)	0.248	(-0.47, 0.97)	0.489	0.814
P_Firmicutes; C_Clostridia; O_Clostridiales; F_Ruminococcaceae; G_Oscillospira	0.201	(-0.18, 0.59)	0.016	(-0.42, 0.45)	0.510	0.814
P_Firmicutes; C_Clostridia; O_Clostridiales; F_Lachnospiraceae; G_Blautia	-0.020	(-0.12, 0.08)	-0.066	(-0.18, 0.05)	0.532	0.814
P_Firmicutes; C_Bacilli; O_Lactobacillales; F_Enterococcaceae; G_Enterococcus	0.209	(-0.41, 0.83)	0.486	(-0.21, 1.18)	0.534	0.814
P_Firmicutes; C_Clostridia; O_Clostridiales; F_Lachnospiraceae; G_Lachnospira	-0.263	(-0.89, 0.36)	-0.010	(-0.71, 0.69)	0.578	0.814

	P_Firmicutes; C_Erysipelotrichi; O_Erysipelotrichales; F_Erysipelotrichaceae; G_Unclassified	0.089	(-0.47, 0.65)	-0.117	(-0.74, 0.51)	0.608	0.814
	P_Firmicutes; C_Clostridia; O_Clostridiales; F_Ruminococcaceae; G_Faecalibacterium	0.176	(0.09, 0.26)	0.146	(0.05, 0.24)	0.616	0.814
	P_Bacteroidetes; C_Bacteroidia; O_Bacteroidales; F_Porphyromonadaceae; G_Parabacteroides	-0.192	(-0.77, 0.38)	0.016	(-0.63, 0.66)	0.625	0.814
	P_Firmicutes; C_Clostridia; O_Clostridiales; F_Lachnospiraceae; G_Coprococcus	0.058	(-0.03, 0.15)	0.026	(-0.08, 0.13)	0.636	0.814
	P_Actinobacteria; C_Coriobacteriia; O_Coriobacteriales; F_Coriobacteriaceae; G_Collinsella	0.325	(-0.04, 0.69)	0.202	(-0.21, 0.61)	0.638	0.814
	P_Firmicutes; C_Clostridia; O_Clostridiales; F_Clostridiaceae; G_Unclassified	0.021	(-0.68, 0.72)	0.261	(-0.53, 1.05)	0.640	0.814
	P_Firmicutes; C_Clostridia; O_Clostridiales; F_Lachnospiraceae; G_Dorea	0.008	(-0.08, 0.09)	-0.017	(-0.11, 0.08)	0.677	0.836
	P_Actinobacteria; C_Actinobacteria; O_Bifidobacteriales; F_Bifidobacteriaceae; G_Bifidobacterium	0.013	(-0.5, 0.53)	0.163	(-0.42, 0.75)	0.699	0.839
	P_Bacteroidetes; C_Bacteroidia; O_Bacteroidales; F_Barnesiellaceae; G_unclassified	-0.097	(-0.4, 0.21)	-0.036	(-0.38, 0.31)	0.787	0.918
	P_Firmicutes; C_Clostridia; O_Clostridiales; F__Unclassified; G__Unclassified	0.074	(-0.17, 0.32)	0.042	(-0.23, 0.31)	0.855	0.967
	P_Firmicutes; C_Clostridia; O_Clostridiales; F_Ruminococcaceae; G_Unclassified	0.182	(-0.24, 0.6)	0.230	(-0.24, 0.7)	0.875	0.967
	P_Firmicutes; C_Clostridia; O_Clostridiales; F_Lachnospiraceae; G_Unclassified	-0.052	(-0.25, 0.14)	-0.070	(-0.29, 0.15)	0.902	0.971
	P_Firmicutes; C_Clostridia; O_Clostridiales; F_Clostridiaceae; G_Clostridium	-0.188	(-0.64, 0.26)	-0.182	(-0.69, 0.32)	0.984	1.000
	P_Bacteroidetes; C_Bacteroidia; O_Bacteroidales; F_Odoribacteraceae; G_Odoribacter	0.130	(-0.3, 0.56)	0.127	(-0.36, 0.61)	0.992	1.000
	P_Firmicutes; C_Bacilli; O_Lactobacillales; F_Streptococcaceae; G_Streptococcus	0.161	(-0.4, 0.73)	0.161	(-0.47, 0.79)	1.000	1.000
OTU	P_Firmicutes; C_Clostridia; O_Clostridiales; F_Ruminococcaceae; G_Unclassified; S_unclassified	-0.083	(-0.26, 0.1)	0.327	(0.13, 0.53)	**0.006**	0.318
	P_Firmicutes; C_Clostridia; O_Clostridiales; F_Ruminococcaceae; G_Ruminococcus; S_bromii	-0.119	(-0.33, 0.09)	0.252	(0.02, 0.49)	**0.026**	0.649
	P_Firmicutes; C_Clostridia; O_Clostridiales; F_Veillonellaceae; G_Phascolarctobacterium; S_unclassified	-0.211	(-0.49, 0.07)	0.250	(-0.06, 0.56)	**0.035**	0.649
	P_Firmicutes; C_Clostridia; O_Clostridiales; F_Lachnospiraceae; G_Lachnobacterium; S_unclassified	0.461	(-0.15, 1.07)	-0.313	(-1, 0.37)	**0.097**	0.785
	P_Bacteroidetes; C_Bacteroidia; O_Bacteroidales; F_Porphyromonadaceae; G_Parabacteroides; S_distasonis	-0.699	(-1.19, -0.2)	-0.114	(-0.67, 0.44)	0.124	0.785
	P_Firmicutes; C_Clostridia; O_Clostridiales; F_Veillonellaceae; G_Dialister; S_unclassified	0.164	(-0.21, 0.54)	-0.236	(-0.65, 0.18)	0.149	0.785
	P_Bacteroidetes; C_Bacteroidia; O_Bacteroidales; F_Prevotellaceae; G_Prevotella; S_copri	0.103	(-0.14, 0.35)	-0.175	(-0.45, 0.1)	0.165	0.785

P_Firmicutes; C_Erysipelotrichi; O_Erysipelotrichales; F_Erysipelotrichaceae; G_Clostridium; S_spiroforme	-0.005	(-0.28, 0.27)	0.286	(-0.02, 0.59)	0.167	0.785
P_Bacteroidetes; C_Bacteroidia; O_Bacteroidales; F_Bacteroidaceae; G_Bacteroides; S_ovatus	-0.026	(-0.42, 0.37)	0.386	(-0.06, 0.83)	0.175	0.785
P_Firmicutes; C_Clostridia; O_Clostridiales; F_Lachnospiraceae; G_Ruminococcus; S_unclassified	0.087	(-0.33, 0.5)	-0.339	(-0.8, 0.13)	0.180	0.785
P_Firmicutes; C_Clostridia; O_Clostridiales; F_Unclassified; G_Unclassified; S_unclassified	-0.132	(-0.52, 0.25)	0.241	(-0.19, 0.67)	0.191	0.785
P_Bacteroidetes; C_Bacteroidia; O_Bacteroidales; F_Bacteroidaceae; G_Bacteroides; S_unclassified	-0.139	(-0.27, -0.01)	-0.021	(-0.17, 0.13)	0.223	0.785
P_Actinobacteria; C_Actinobacteria; O_Bifidobacteriales; F_Bifidobacteriaceae; G_Bifidobacterium; S_unclassified	-0.128	(-0.62, 0.36)	0.312	(-0.24, 0.86)	0.226	0.785
P_Firmicutes; C_Clostridia; O_Clostridiales; F_Mogibacteriaceae; G_Unclassified; S_unclassified	0.313	(-0.04, 0.67)	0.641	(0.24, 1.04)	0.235	0.785
P_Firmicutes; C_Clostridia; O_Clostridiales; F_Lachnospiraceae; G_Dorea; S_unclassified	0.034	(-0.07, 0.14)	-0.053	(-0.17, 0.06)	0.259	0.785
P_Bacteroidetes; C_Bacteroidia; O_Bacteroidales; F_Rikenellaceae; G_Unclassified; S_unclassified	0.029	(-0.33, 0.39)	0.332	(-0.08, 0.74)	0.261	0.785
P_Firmicutes; C_Clostridia; O_Clostridiales; F_Lachnospiraceae; G_Roseburia; S_unclassified	0.242	(-0.1, 0.59)	-0.037	(-0.42, 0.35)	0.269	0.785
P_Firmicutes; C_Clostridia; O_Clostridiales; F_Ruminococcaceae; G_Gemmiger; S_formicilis	0.006	(-0.08, 0.09)	0.076	(-0.02, 0.17)	0.272	0.785
P_Firmicutes; C_Erysipelotrichi; O_Erysipelotrichales; F_Erysipelotrichaceae; G_Eubacterium; S_biforme	0.035	(-0.21, 0.28)	-0.161	(-0.44, 0.12)	0.278	0.785
P_Firmicutes; C_Clostridia; O_Clostridiales; F_Christensenellaceae; G_Unclassified; S_unclassified	-0.109	(-0.62, 0.41)	0.287	(-0.29, 0.86)	0.293	0.785
P_Firmicutes; C_Clostridia; O_Clostridiales; F_Ruminococcaceae; G_Ruminococcus; S_unclassified	-0.173	(-0.4, 0.05)	-0.003	(-0.26, 0.25)	0.313	0.785
P_Firmicutes; C_Clostridia; O_Clostridiales; F_Lachnospiraceae; G_Blautia; S_obeum	-0.073	(-0.53, 0.39)	0.263	(-0.25, 0.77)	0.318	0.785
P_Proteobacteria; C_Betaproteobacteria; O_Burkholderiales; F_Alcaligenaceae; G_Sutterella; S_unclassified	0.093	(-0.41, 0.6)	0.485	(-0.09, 1.06)	0.332	0.785
P_Firmicutes; C_Clostridia; O_Clostridiales; F_Lachnospiraceae; G_Roseburia; S_unclassified	0.175	(-0.25, 0.6)	-0.130	(-0.61, 0.35)	0.336	0.785
P_Firmicutes; C_Clostridia; O_Clostridiales; F_Lachnospiraceae; G_Roseburia; S_faecis	0.155	(-0.21, 0.52)	-0.086	(-0.49, 0.32)	0.372	0.833
P_Firmicutes; C_Clostridia; O_Clostridiales; F_Lachnospiraceae; G_Unclassified; S_unclassified	0.187	(0.09, 0.29)	0.125	(0.01, 0.24)	0.391	0.842
P_Firmicutes; C_Clostridia; O_Clostridiales; F_Lachnospiraceae; G_Ruminococcus; S_torques	-0.116	(-0.73, 0.49)	0.248	(-0.44, 0.93)	0.421	0.842
P_Actinobacteria; C_Coriobacteriia; O_Coriobacteriales; F_Coriobacteriaceae; G_Collinsella; S_aerofaciens	0.335	(-0.04, 0.71)	0.120	(-0.3, 0.54)	0.426	0.842
P_Bacteroidetes; C_Bacteroidia; O_Bacteroidales; F_Bacteroidaceae; G_Bacteroides__unclassified	-0.126	(-0.48, 0.23)	0.061	(-0.34, 0.46)	0.467	0.842

P_Firmicutes; C_Clostridia; O_Clostridiales; F_Clostridiaceae; G_SMB53; S_unclassified	0.462	(-0.16, 1.09)	0.139	(-0.56, 0.84)	0.477	0.842
P_Bacteroidetes; C_Bacteroidia; O_Bacteroidales; F_Porphyromonadaceae; G_Parabacteroides; S_unclassified	0.251	(-0.21, 0.71)	0.019	(-0.49, 0.53)	0.485	0.842
P_Firmicutes; C_Clostridia; O_Clostridiales; F_Lachnospiraceae; G_Anaerostipes; S_unclassified	-0.075	(-0.72, 0.57)	0.248	(-0.47, 0.97)	0.489	0.842
P_Firmicutes; C_Clostridia; O_Clostridiales; F_Ruminococcaceae; G_Oscillospira; S_unclassified	0.201	(-0.18, 0.59)	0.016	(-0.42, 0.45)	0.510	0.842
P_Firmicutes; C_Clostridia; O_Clostridiales; F_Lachnospiraceae; G_Blautia; S_unclassified	-0.019	(-0.11, 0.08)	-0.063	(-0.17, 0.04)	0.521	0.842
P_Bacteroidetes; C_Bacteroidia; O_Bacteroidales; F_Bacteroidaceae; G_Bacteroides; S_uniformis	-0.086	(-0.71, 0.54)	0.187	(-0.52, 0.89)	0.555	0.842
P_Firmicutes; C_Clostridia; O_Clostridiales; F_Ruminococcaceae; G_Ruminococcus; S_unclassified	0.157	(-0.49, 0.81)	-0.105	(-0.83, 0.62)	0.576	0.842
P_Firmicutes; C_Clostridia; O_Clostridiales; F_Lachnospiraceae; G_Lachnospira; S_unclassified	-0.263	(-0.89, 0.36)	-0.010	(-0.71, 0.69)	0.578	0.842
P_Firmicutes; C_Clostridia; O_Clostridiales; F_Lachnospiraceae; G_Coprococcus; S_catus	0.238	(-0.09, 0.56)	0.106	(-0.26, 0.47)	0.578	0.842
P_Firmicutes; C_Clostridia; O_Clostridiales; F_Lachnospiraceae; G_Ruminococcus; S_gnavus	0.166	(0.01, 0.32)	0.106	(-0.07, 0.28)	0.598	0.842
P_Firmicutes; C_Erysipelotrichi; O_Erysipelotrichales; F_Erysipelotrichaceae; G_Unclassified; S_unclassified	0.089	(-0.47, 0.65)	-0.117	(-0.74, 0.51)	0.608	0.842
P_Firmicutes; C_Clostridia; O_Clostridiales; F_Ruminococcaceae; G_Faecalibacterium; S_prausnitzii	0.176	(0.09, 0.26)	0.146	(0.05, 0.24)	0.616	0.842
P_Firmicutes; C_Clostridia; O_Clostridiales; F_Clostridiaceae; G_Unclassified; S_unclassified	0.021	(-0.68, 0.72)	0.261	(-0.53, 1.05)	0.640	0.853
P_Firmicutes; C_Clostridia; O_Clostridiales; F_Lachnospiraceae; G_Coprococcus; S_eutactus	0.184	(-0.18, 0.55)	0.079	(-0.33, 0.49)	0.689	0.894
P_Firmicutes; C_Bacilli; O_Lactobacillales; F_Streptococcaceae; G_Streptococcus; S_unclassified	0.001	(-0.62, 0.62)	0.170	(-0.52, 0.86)	0.706	0.894
P_Bacteroidetes; C_Bacteroidia; O_Bacteroidales; F_Bacteroidaceae; G_Bacteroides; S_caccae	-0.294	(-0.86, 0.27)	-0.154	(-0.79, 0.48)	0.734	0.894
P_Firmicutes; C_Clostridia; O_Clostridiales; F_Lachnospiraceae; G_Coprococcus; S_unclassified	0.011	(-0.11, 0.13)	-0.018	(-0.15, 0.11)	0.735	0.894
P_Bacteroidetes; C_Bacteroidia; O_Bacteroidales; F_Barnesiellaceae; G_Unclassified; S_unclassified	-0.097	(-0.4, 0.21)	-0.036	(-0.38, 0.31)	0.787	0.920
P_Firmicutes; C_Clostridia; O_Clostridiales; F_Ruminococcaceae; G_Ruminococcus; S_callidus	0.132	(-0.23, 0.49)	0.201	(-0.2, 0.6)	0.789	0.920
P_Firmicutes; C_Clostridia; O_Clostridiales; F_Clostridiaceae; G_Clostridium; S_unclassified	-0.166	(-0.67, 0.34)	-0.078	(-0.65, 0.49)	0.817	0.934
P_Firmicutes; C_Clostridia; O_Clostridiales; F_Unclassified; G_unclassified; S_unclassified	0.074	(-0.17, 0.32)	0.042	(-0.23, 0.31)	0.855	0.935
P_Firmicutes; C_Clostridia; O_Clostridiales; F_Ruminococcaceae; G_Unclassified; S_unclassified	0.182	(-0.24, 0.6)	0.230	(-0.24, 0.7)	0.875	0.935

P_Actinobacteria; C_Actinobacteria; O_Bifidobacteriales; F_Bifidobacteriaceae; G_Bifidobacterium; S_longum	-0.020	(-0.55, 0.51)	0.038	(-0.56, 0.63)	0.879	0.935
P_Actinobacteria; C_Actinobacteria; O_Bifidobacteriales; F_Bifidobacteriaceae; G_Bifidobacterium; S_adolescentis	0.447	(-0.06, 0.96)	0.397	(-0.18, 0.98)	0.899	0.935
P_Firmicutes; C_Clostridia; O_Clostridiales; F_Lachnospiraceae; G_Unclassified; S_unclassified	-0.052	(-0.25, 0.14)	-0.070	(-0.29, 0.15)	0.902	0.935
P_Firmicutes; C_Clostridia; O_Clostridiales; F_Lachnospiraceae; G_Dorea; S_formicigenerans	0.161	(-0.15, 0.47)	0.169	(-0.17, 0.51)	0.973	0.991
P_Bacteroidetes; C_Bacteroidia; O_Bacteroidales; F_Odoribacteraceae; G_Odoribacter; S_unclassified	0.130	(-0.3, 0.56)	0.127	(-0.36, 0.61)	0.992	0.992

[a] Mean change values, 95% confidence intervals, and p-values were calculated as the least squares means from linear regression models adjusted for baseline values. Bold values indicate a raw p-value that was stastistically significant (p<0.05) or borderline significant (p<0.10).

[b] q-values were calculated using the Benjamini-Hochberg method

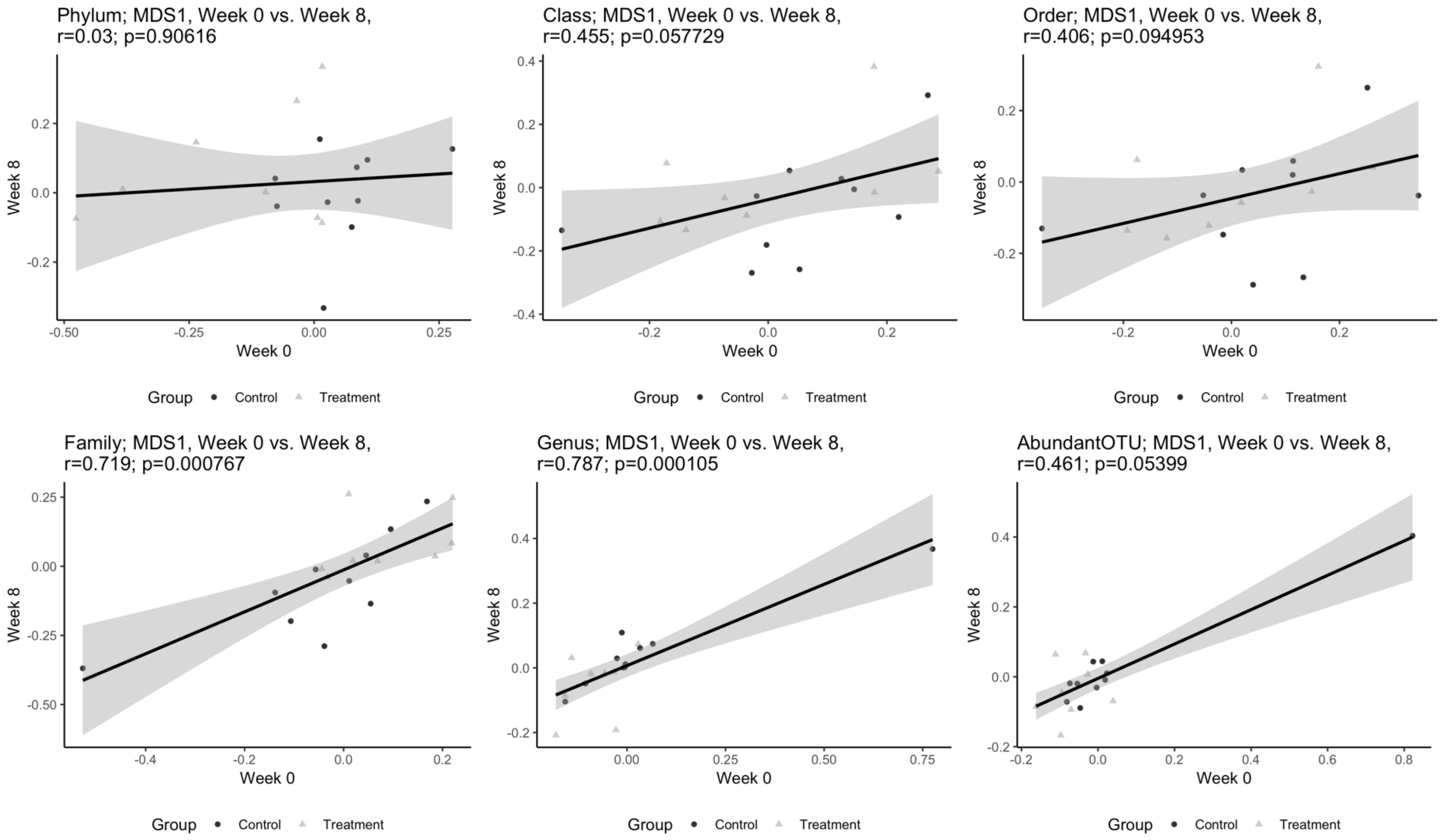

Figure S1: Correlations between baseline (week 0, *x*-axis) and week 8 (*y*-axis) for the first multidimensional scaling axis (MDS) in PCoA analysis at each taxa level. Black line indicates the estimated linear trend based on a linear regression model (method="lm"). P-values and correlation coefficients (r) were calculated by Spearman correlation. Ordination was based on Bray-Curtis dissimilarity.

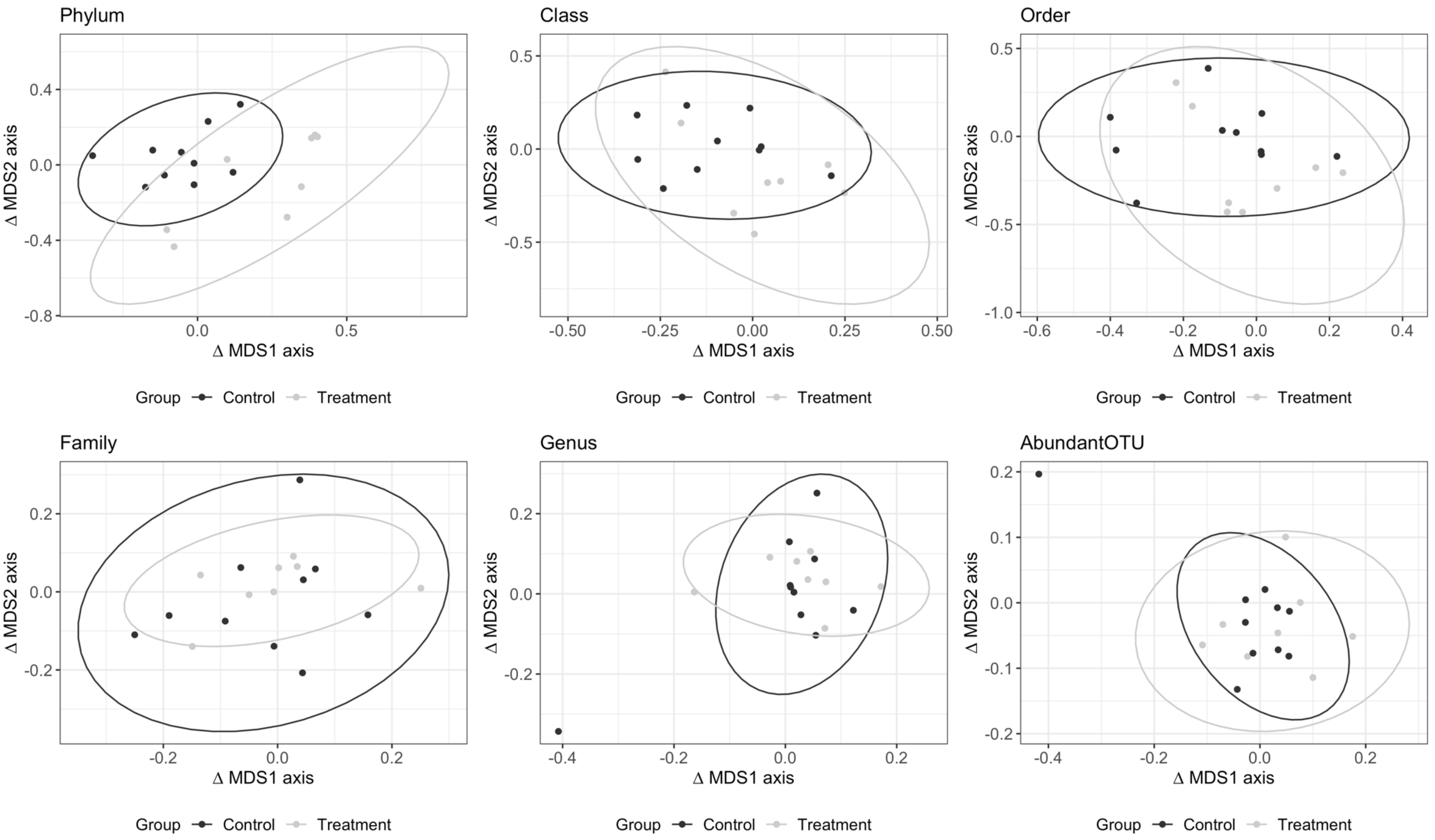

Figure S2: Multidimensional scaling (MDS) ordination at each taxonomic level. Ordination based on Bray-Curtis dissimilarity. Plots show that there were no significant differences in change values for the first and second MDS axes between groups (Table S4).
